# Supplementary material for: Fingerprints of the COVID-19 economic downturn and recovery on ozone anomalies at high-elevation sites in North America and western Europe
Source: Atmos Chem Phys. Author manuscript; Available in PMC 2025 Jun 21. (PMC12181938; doi:10.5194/acp-23-15693-2023)
Supplement: Supplement PDF [file NIHMS2067795-supplement-Supplement_PDF.pdf]

Supplement of Atmos. Chem. Phys., 23, 15693–15709, 2023
https://doi.org/10.5194/acp-23-15693-2023-supplement
© Author(s) 2023. CC BY 4.0 License.

Atmospheric Chemistry and Physics
Open Access

Supplement of

Fingerprints of the COVID-19 economic downturn and recovery on ozone anomalies at high-elevation sites in North America and western Europe

Davide Putero et al.

Correspondence to: Davide Putero (d.putero@isac.cnr.it)

The copyright of individual parts of the supplement might differ from the article licence.

S1. Zugspitze data homogenization

Similarly to Cooper et al. (2020) and Cristofanelli et al. (2020), the Zugspitze data were homogenized to take into account the different O_3 measurement activities that were carried out during the years, i.e.: O_3 measurements carried out by the Fraunhofer Institute for Atmospheric Environment Research (IMK-IFU) at the summit of Mt. Zugspitze (ZUG, 2962 m a.s.l.) from 1978 to 2010, and the O_3 measurements from the German Federal Environmental Agency (UBA) at the Schneefernerhaus station (ZSF, 2669 m a.s.l.) from 2001. The time series at ZUG (2000–2010) and ZSF (2011–2021) were merged, taking into account the bias due to the lower elevation of ZSF with respect to ZUG. To homogenize the two time series, the simultaneous observations at ZUG and ZSF were compared from 2002 to 2010, and the average differences between monthly nighttime (00–04 UTC) data were calculated, resulting in a mean bias of 1.32 ppb. This average bias was then subtracted from the ZUG time series, to obtain a unique consistent time series, and to avoid artificial discontinuities due to the different sampling locations. The merged time series obtained the ZSF acronym in the paper, for continuity with the measurements currently carried out at the site.

Table S1: Decadal ozone trends (50th percentile) for the 41 high-elevation sites used in this study, with different periods considered for trend calculation (i.e., 2000–2019, 2000–2020, and 2000–2021, or shorter periods when data back to 2000 were not available). For station acronyms please refer to Table 1.

Site	Full record, ending in 2019 (ppb/decade)	Full record, ending in 2020 (ppb/decade)	Full record, ending in 2021 (ppb/decade)
GNP	1.48 [± 1.42], $p = 0.04$	1.40 [± 1.32], $p = 0.04$	1.30 [± 1.10], $p = 0.02$
YEL	−1.45 [± 0.97], $p < 0.01$	−1.42 [± 0.99], $p < 0.01$	−1.54 [± 0.90], $p < 0.01$
MBO	3.09 [± 2.19], $p = 0.01$	2.47 [± 2.08], $p = 0.02$	2.82 [± 2.23], $p = 0.01$
GTP	−3.45 [± 3.12], $p = 0.03$	−2.55 [± 2.78], $p = 0.07$	−1.28 [± 2.74], $p = 0.35$
WNC	−1.54 [± 1.62], $p = 0.06$	−0.96 [± 1.78], $p = 0.28$	−0.67 [± 1.54], $p = 0.39$
CRA	−1.90 [± 1.61], $p = 0.02$	−1.74 [± 1.57], $p = 0.03$	−1.21 [± 1.34], $p = 0.03$
PND	−1.33 [± 0.77], $p < 0.01$	−1.32 [± 0.73], $p < 0.01$	−1.38 [± 0.74], $p < 0.01$
CNR	−1.69 [± 0.96], $p < 0.01$	−1.72 [± 0.96], $p < 0.01$	−1.65 [± 1.05], $p < 0.01$
LAV	−1.50 [± 0.93], $p < 0.01$	−1.63 [± 0.98], $p < 0.01$	−1.28 [± 0.82], $p < 0.01$
DIN	0.68 [± 2.43], $p = 0.57$	−0.07 [± 2.53], $p = 0.95$	−0.27 [± 2.20], $p = 0.81$
RAN	−1.75 [± 3.54], $p = 0.32$	−2.52 [± 3.33], $p = 0.13$	−2.30 [± 2.49], $p = 0.07$
GBN	−0.35 [± 0.87], $p = 0.43$	−0.27 [± 0.80], $p = 0.50$	−0.26 [± 0.80], $p = 0.51$
GTH	−1.73 [± 0.95], $p < 0.01$	−1.67 [± 1.02], $p < 0.01$	−1.56 [± 0.91], $p < 0.01$
CAN	−1.51 [±1.07], $p < 0.01$	−1.77 [±1.01], $p < 0.01$	−1.87 [± 0.98], $p < 0.01$
MEV	−0.13 [± 1.17], $p = 0.82$	−0.19 [± 1.16], $p = 0.74$	−0.22 [± 1.01], $p = 0.66$
ZIO	−2.52 [± 1.12], $p < 0.01$	−2.47 [± 1.16], $p < 0.01$	−2.11 [± 0.97], $p < 0.01$
GRC	−2.09 [± 1.08], $p < 0.01$	−2.12 [± 1.09], $p < 0.01$	−2.06 [± 0.90], $p < 0.01$
PFN	−2.07 [± 1.11], $p < 0.01$	−2.10 [± 1.06], $p < 0.01$	−1.86 [± 0.91], $p < 0.01$
CNM	−1.21 [± 1.18], $p = 0.04$	−1.32 [± 1.15], $p = 0.02$	−1.13 [± 1.13], $p = 0.05$
RMN	0.22 [± 0.83], $p = 0.60$	0.16 [± 0.80], $p = 0.69$	0.12 [± 0.81], $p = 0.77$
YOS	−3.18 [± 1.66], $p < 0.01$	−3.13 [± 1.66], $p < 0.01$	−2.65 [± 1.68], $p < 0.01$
SQA	−3.05 [± 1.82], $p < 0.01$	−2.92 [± 1.65], $p < 0.01$	−2.51 [± 1.84], $p = 0.01$
JOT	−1.66 [± 1.60], $p = 0.04$	−1.78 [± 1.63], $p = 0.03$	−1.87 [± 1.57], $p = 0.02$
WFM	−2.68 [± 1.58], $p < 0.01$	−2.42 [± 1.42], $p < 0.01$	−2.00 [± 1.47], $p = 0.01$
SHN	−2.49 [± 1.94], $p = 0.01$	−2.83 [± 2.25], $p = 0.01$	−2.84 [± 2.03], $p = 0.01$
PNF	−3.04 [± 1.42], $p < 0.01$	−3.30 [± 1.57], $p < 0.01$	−3.16 [± 1.37], $p < 0.01$

GSM	−4.28 [± 1.50], $p < 0.01$	−4.55 [± 1.63], $p < 0.01$	−4.51 [± 1.49], $p < 0.01$
HPB	−1.43 [± 1.07], $p = 0.01$	−1.26 [± 1.01], $p = 0.01$	−1.41 [± 1.00], $p = 0.01$
ZSF	−0.28 [± 0.68], $p = 0.41$	−0.44 [± 0.69], $p = 0.20$	−0.61 [± 0.68], $p = 0.13$
SNB	−1.23 [± 0.69], $p < 0.01$	−1.32 [± 0.65], $p < 0.01$	−1.41 [± 0.66], $p < 0.01$
JFJ	−0.12 [± 0.78], $p = 0.75$	−0.44 [± 0.97], $p = 0.36$	−0.70 [± 1.04], $p = 0.18$
CMN	−2.08 [± 1.40], $p < 0.01$	−2.23 [± 1.37], $p < 0.01$	−2.47 [± 1.48], $p < 0.01$
SUM	−1.66 [± 1.16], $p < 0.01$	−2.06 [± 1.20], $p < 0.01$	−2.37 [± 1.19], $p < 0.01$
DEN	0.33 [± 1.13], $p = 0.56$	0.27 [± 0.95], $p = 0.57$	0.19 [± 0.82], $p = 0.65$
IZO	0.47 [± 0.92], $p = 0.31$	0.26 [± 0.83], $p = 0.53$	0.17 [± 0.84], $p = 0.68$
PDI	0.92 [± 16.00], $p = 0.91$	−7.80 [± 15.36], $p = 0.31$	−4.79 [± 11.75], $p = 0.42$
MLO	0.42 [± 1.87], $p = 0.65$	0.47 [± 1.63], $p = 0.56$	0.35 [± 1.45], $p = 0.63$
MKN	1.68 [± 3.23], $p = 0.30$	1.02 [± 2.38], $p = 0.39$	1.70 [± 2.45], $p = 0.17$
TLL	2.33 [± 0.70], $p < 0.01$	2.36 [± 0.68], $p < 0.01$	2.26 [± 0.58], $p < 0.01$
DCC	0.97 [± 0.97], $p = 0.07$	0.97 [± 0.90], $p = 0.03$	0.32 [± 1.05], $p = 0.55$
SPO	1.33 [± 0.43], $p < 0.01$	1.44 [± 0.38], $p < 0.01$	1.32 [± 0.41], $p < 0.01$

Table S2: CO_2 emissions variations (expressed in %) from Carbon Monitor (Liu et al., 2020), for EU27 states and United Kingdom emissions, for the different combinations of years 2019, 2020, and 2021, and with focus on MAM and JJA for each comparison. The percentage represents the contribution of each sector to the total change (i.e., "All sectors"), while the percentage in parentheses indicates the sector change in the selected year with respect to the comparison year.

**EU**	**2020 vs 2019**			**2021 vs 2019**			**2021 vs 2020**		
**Sector**	**All**	**MAM**	**JJA**	**All**	**MAM**	**JJA**	**All**	**MAM**	**JJA**
All sectors	−10.9%	−21.8%	−10.4%	−2.7%	−2.9%	−6.3%	+9.2%	+24.2%	+4.5%
Power	−3.8% (−13.5%)	−6.4% (−24.7%)	−2.8% (−9.4%)	−1.3% (−4.6%)	−1.7% (−6.3%)	−2.5% (−8.4%)	+2.8% (10.2%)	+6.1% (24.3%)	+0.3% (1.1%)
Industry	−1.5% (−7.7%)	−4.1% (−20.6%)	−1.6% (−7.7%)	+0.3% (1.4%)	+0.1% (0.3%)	+0.2% (1.2%)	+2.0% (9.8%)	+5.4% (26.4%)	+2.1% (9.6%)
Ground transport	−1.5% (−5.6%)	−6.1% (−22.7%)	−0.5% (−1.7%)	+0.2% (0.6%)	+0.3% (1.2%)	−0.5% (−1.6%)	+1.9% (6.6%)	+8.3% (30.9%)	+0.0% (0.0%)
Residential	−0.4% (−2.0%)	−0.6% (−2.8%)	+0.6% (6.4%)	+1.1% (6.0%)	+2.6% (12.6%)	+0.3% (3.2%)	+1.7% (8.1%)	+4.0% (15.9%)	−0.3% (−3.0%)
Domestic aviation	−0.2% (−48.0%)	−0.4% (−75.0%)	−0.3% (−50.7%)	−0.2% (−31.8%)	−0.3% (−54.7%)	−0.1% (−16.1%)	+0.1% (31.1%)	+0.1% (81.0%)	+0.2% (70.2%)
International aviation	−3.4% (−58.8%)	−4.2% (−72.5%)	−5.6% (−69.7%)	−2.8% (−48.3%)	−4.0% (−68.9%)	−3.7% (−45.5%)	+0.7% (25.5%)	+0.3% (12.8%)	+2.2% (79.7%)

Table S3: CO_2 emissions variations (expressed in %) from Carbon Monitor (Liu et al., 2020), for United States of America emissions, for the different combinations of years 2019, 2020, and 2021, and with focus on MAM and JJA for each comparison. The percentage represents the contribution of each sector to the total change (i.e., "All sectors"), while the percentage in parentheses indicates the sector change in the selected year with respect to the comparison year.

**US**	**2020 vs 2019**			**2021 vs 2019**			**2021 vs 2020**		
**Sector**	**All**	**MAM**	**JJA**	**All**	**MAM**	**JJA**	**All**	**MAM**	**JJA**
All sectors	−10.1%	−17.2%	−8.7%	−4.5%	−5.8%	−0.3%	+6.2%	+13.8%	+9.2%
Power	−3.3% (−10.6%)	−5.3% (−18.5%)	−1.6% (−4.4%)	−1.4% (−4.4%)	−2.9% (−10.2%)	+1.3% (3.5%)	+2.2% (6.9%)	+2.9% (10.3%)	+3.2% (8.2%)
Industry	−0.9% (−4.6%)	−1.7% (−8.4%)	−1.3% (−6.8%)	−0.4% (−2.3%)	−0.3% (−1.6%)	−0.1% (−0.3%)	+0.5% (2.5%)	+1.7% (7.5%)	+1.4% (6.9%)
Ground transport	−3.1% (−9.6%)	−6.8% (−20.0%)	−3.1% (−9.4%)	−1.0% (−3.2%)	−0.7% (−2.1%)	−0.7% (−2.0%)	+2.3% (7.1%)	+7.4% (22.4%)	+2.7% (8.1%)
Residential	−1.0% (−8.1%)	−1.0% (−8.0%)	−0.2% (−4.5%)	−0.8% (−6.8%)	−0.9% (−7.0%)	−0.2% (−3.2%)	+0.2% (1.4%)	+0.1% (1.0%)	+0.1% (1.4%)
Domestic aviation	−1.0% (−30.4%)	−1.6% (−43.6%)	−1.4% (−39.0%)	−0.3% (−8.5%)	−0.4% (−11.9%)	−0.1% (−2.4%)	+0.8% (31.3%)	+1.4% (56.3%)	+1.4% (59.8%)
International aviation	−0.7% (−45.8%)	−0.9% (−56.0%)	−1.1% (−62.1%)	−0.5% (−33.9%)	−0.6% (−38.4%)	−0.6% (−35.9%)	+0.2% (22.0%)	+0.4% (40.2%)	+0.5% (69.1%)

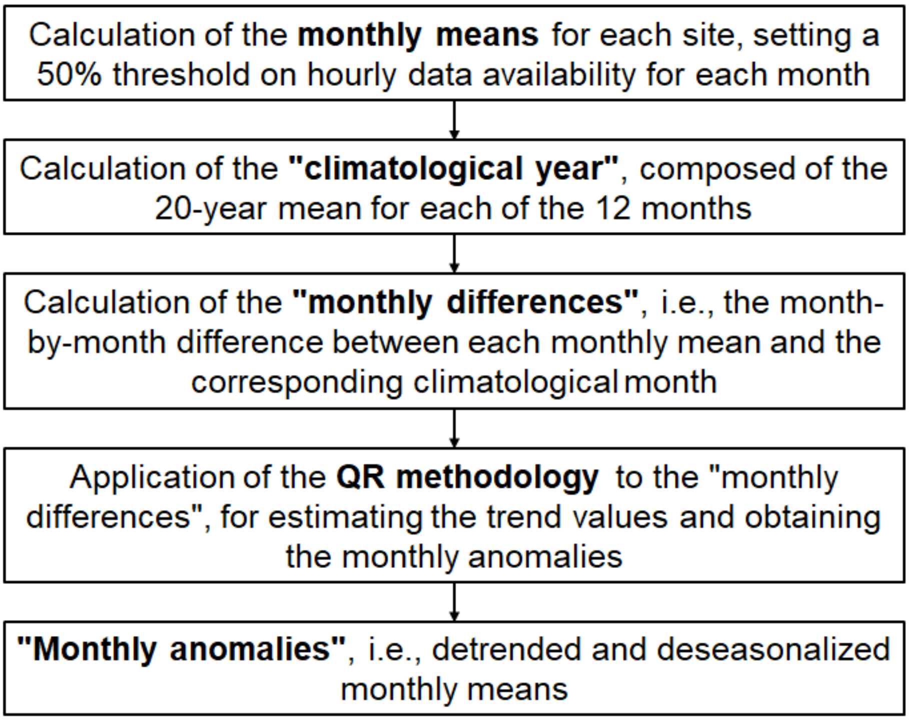

Figure S1. Flowchart indicating the steps followed for the calculation of the monthly O_3 anomalies, as explained in Sect. 2.1.2.

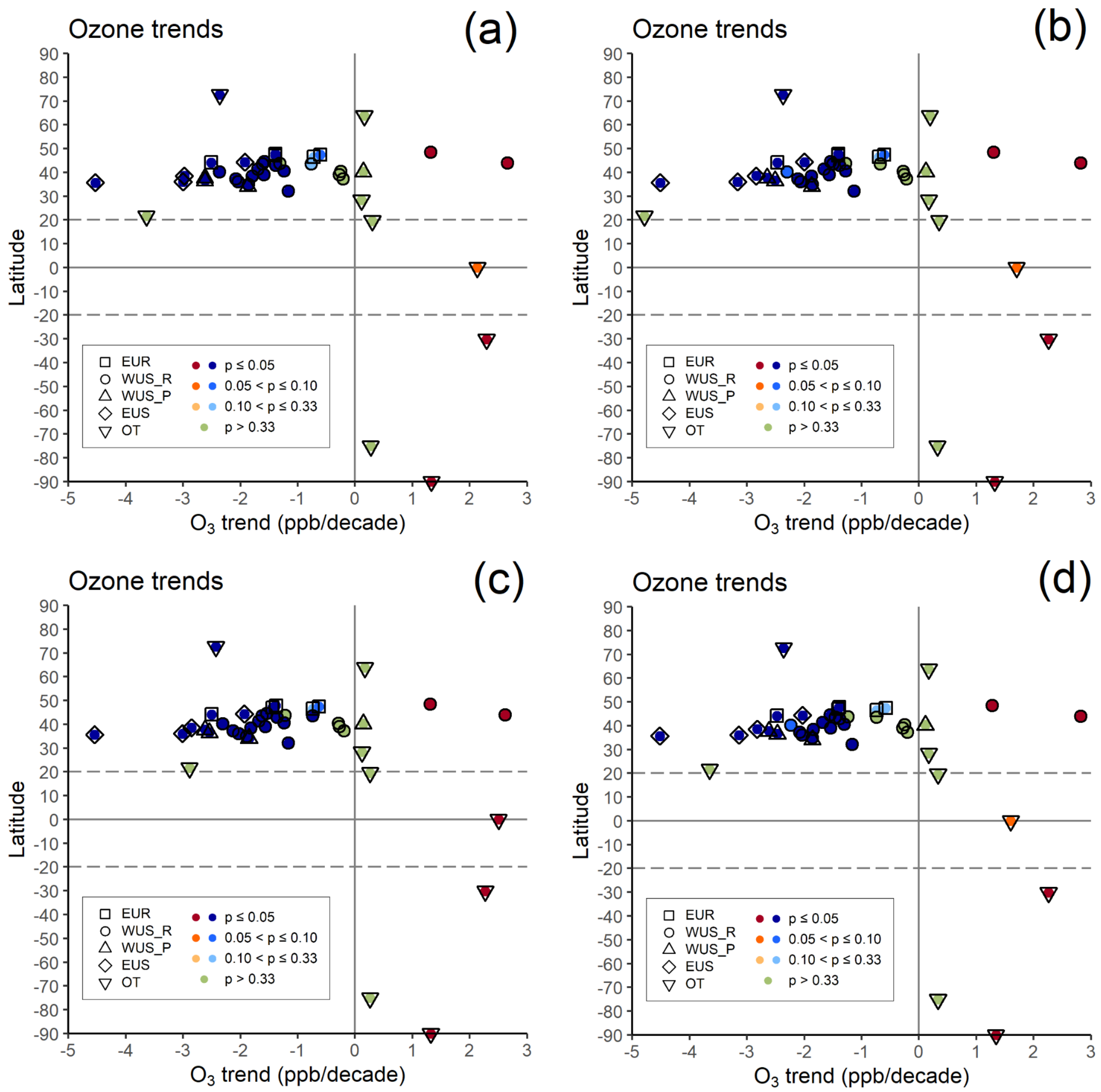

Figure S2: Same as Fig. 2, but with using different thresholds for data availability, i.e., using: (a) a threshold of 66% on hourly data availability, (b) a threshold of 50% on hourly data availability, as in Fig. 2, (c) a threshold of 66% on daily data availability, where the 66% limit is also used for computing daily means, and (d) same as (c), but considering a 50% threshold.

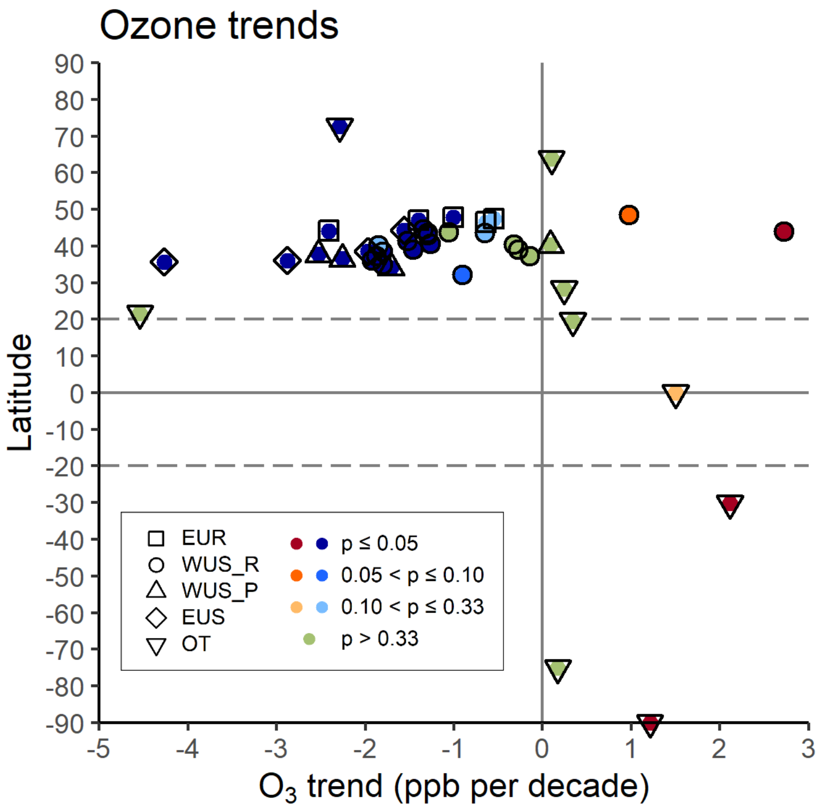

Figure S3. Same as Fig. 2, but in this case the "climatological year" for obtaining the O_3 anomalies was derived by median averaging.

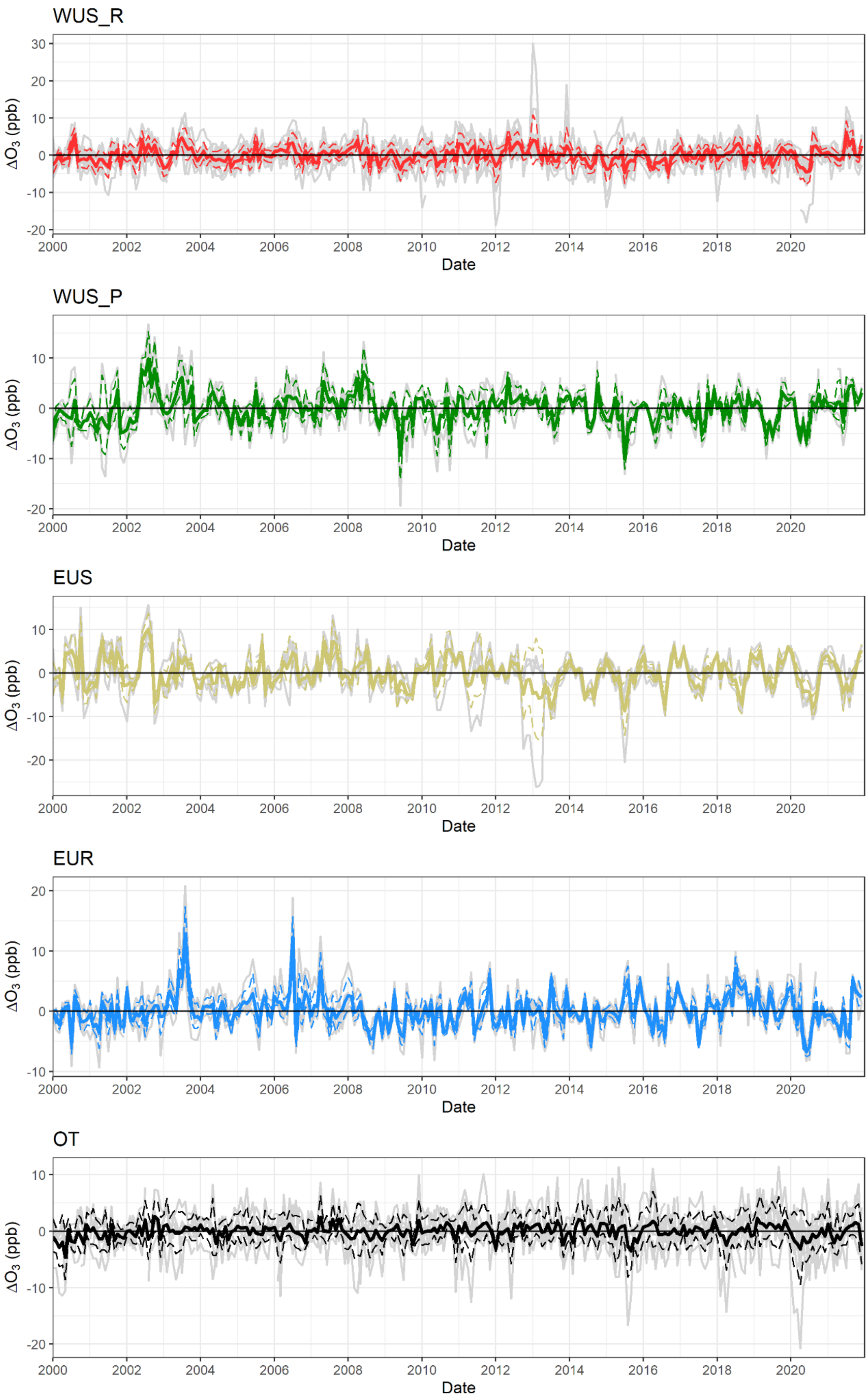

Figure S4: Time series of the monthly O_3 anomalies for the different sites, grouped by region. The thick colored line represents the average time series for each specific region, together with ±1 standard deviation (dashed lines).

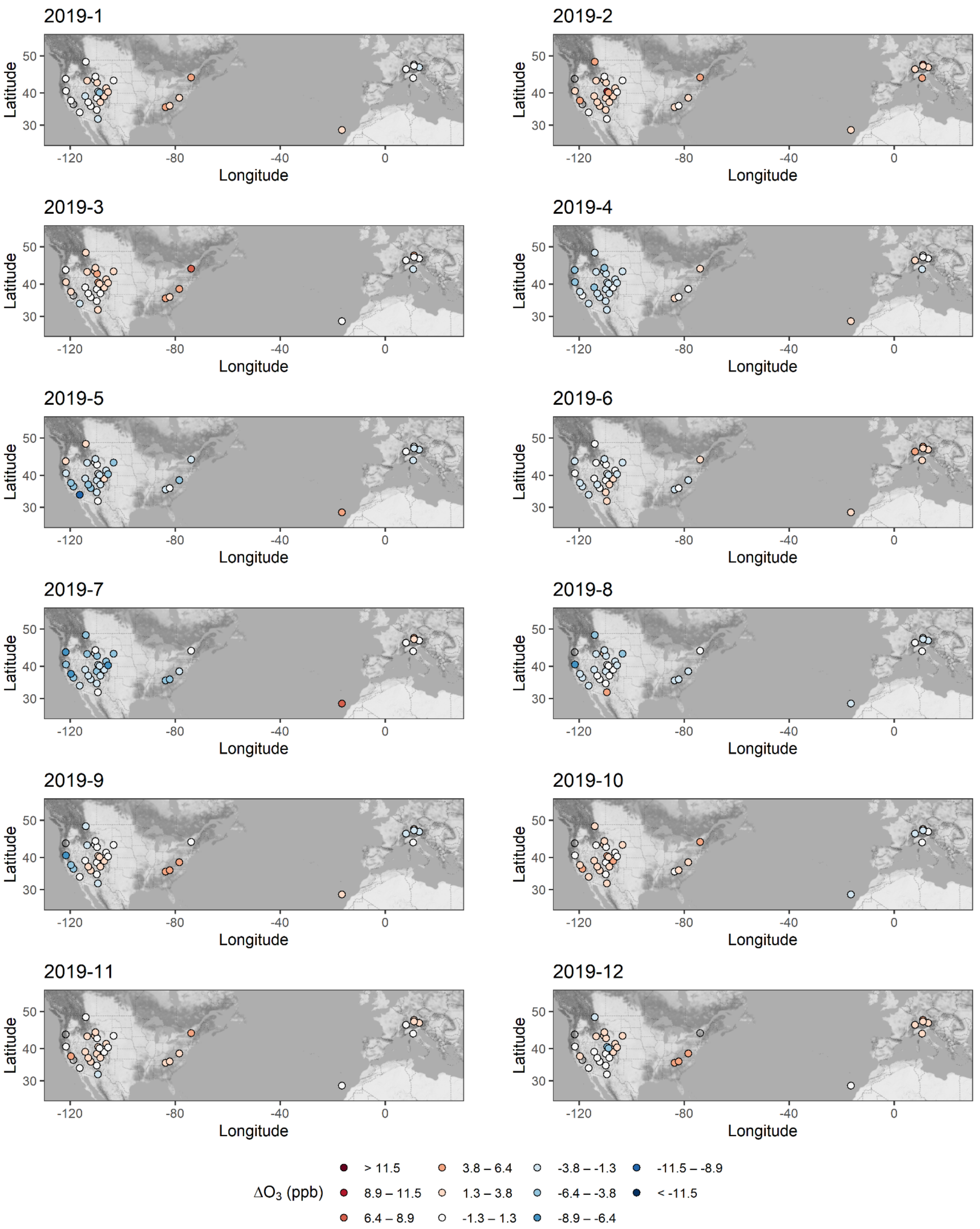

Figure S5. Spatial distribution of the anomalies for the different months of 2019, for sites in North America and Western Europe.

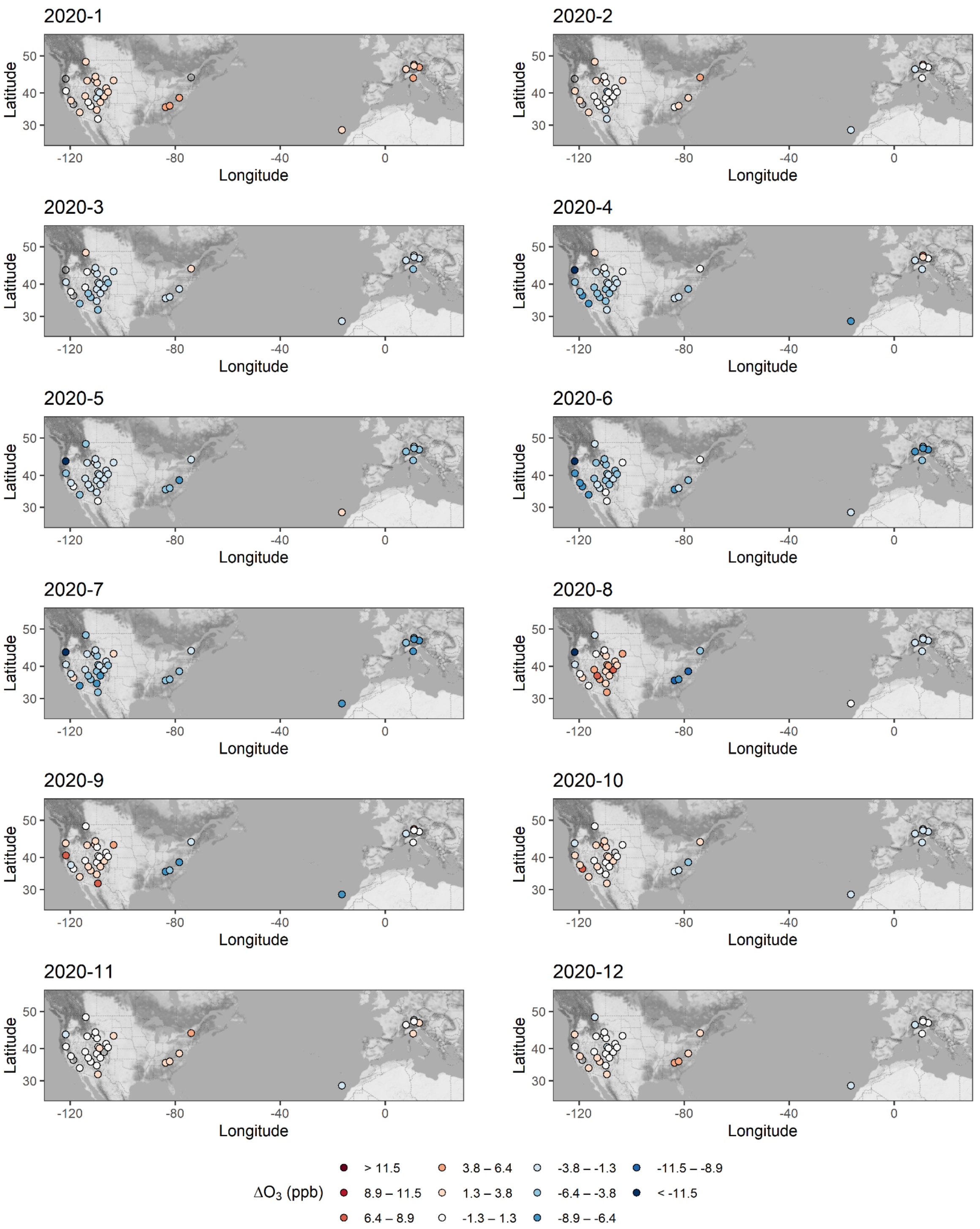

Figure S6. Same as Fig. S5, but for 2020.

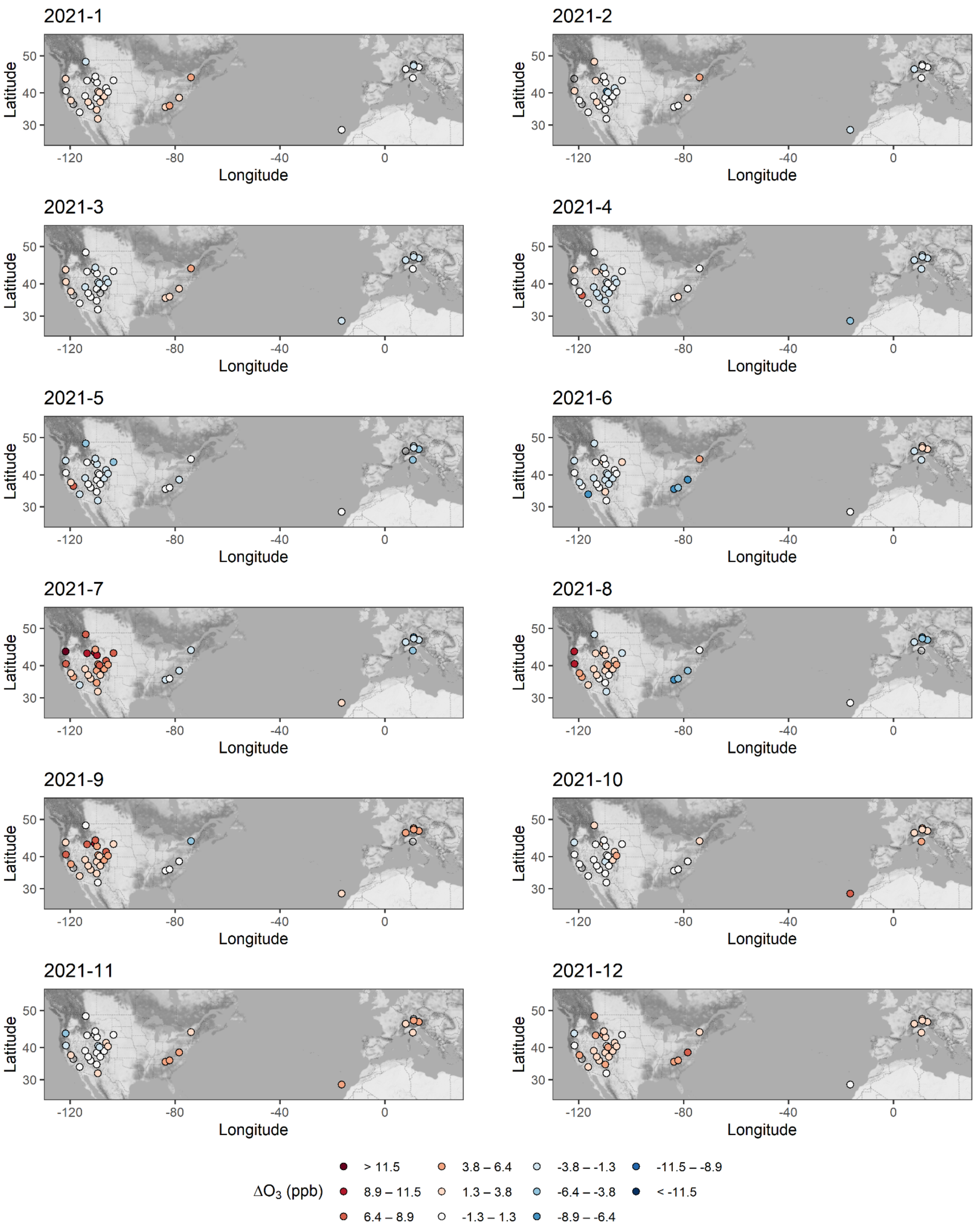

Figure S7. Same as Fig. S5, but for 2021.

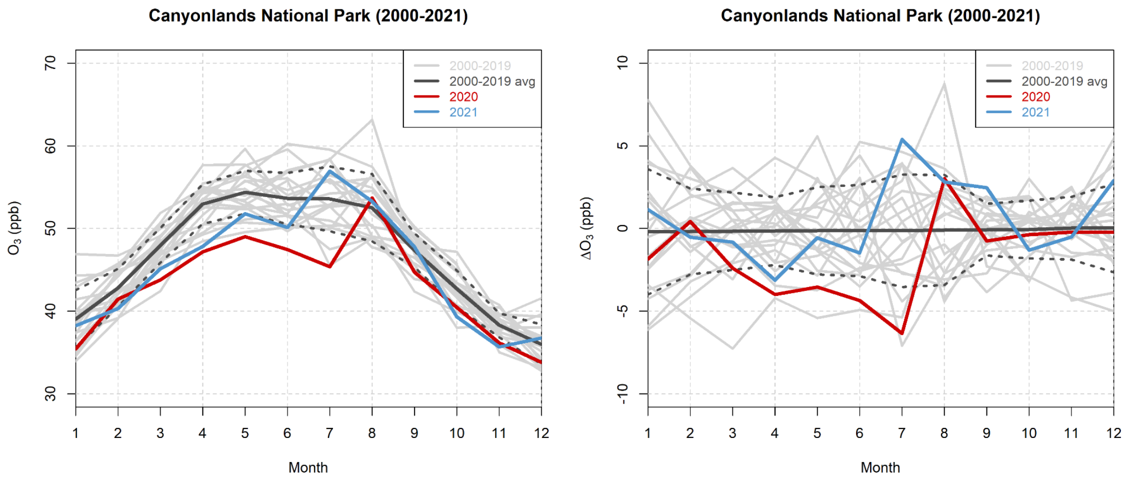

Figure S8: Annual variability of the O_3 monthly averages (left) and anomalies (right) at CAN. The gray lines indicate the single years from 2000 to 2019, the black line is the 2000–2019 climatology (together with ±1 standard deviation, dotted lines), and the red and blue lines indicate 2020 and 2021.

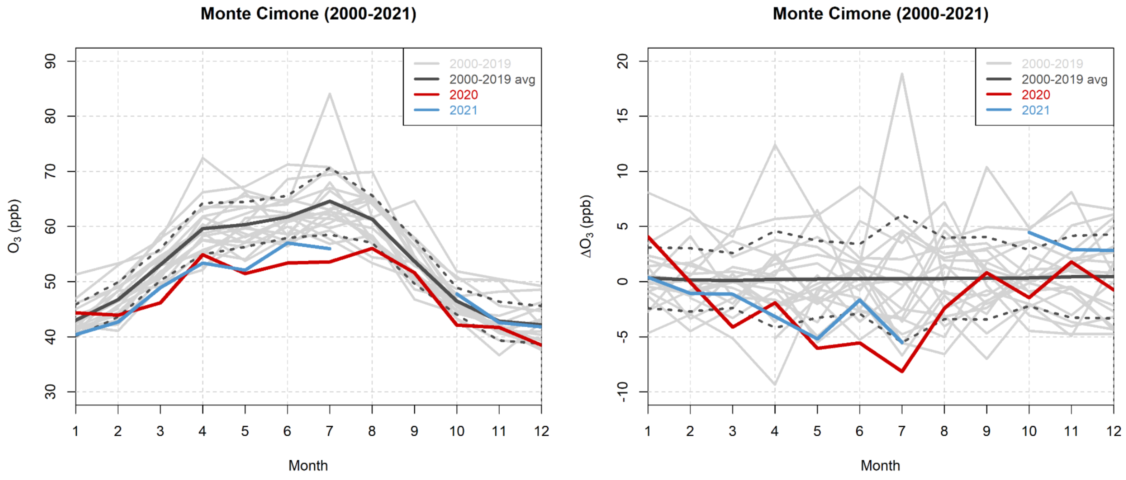

Figure S9. Same as Fig. S8, but for CMN.

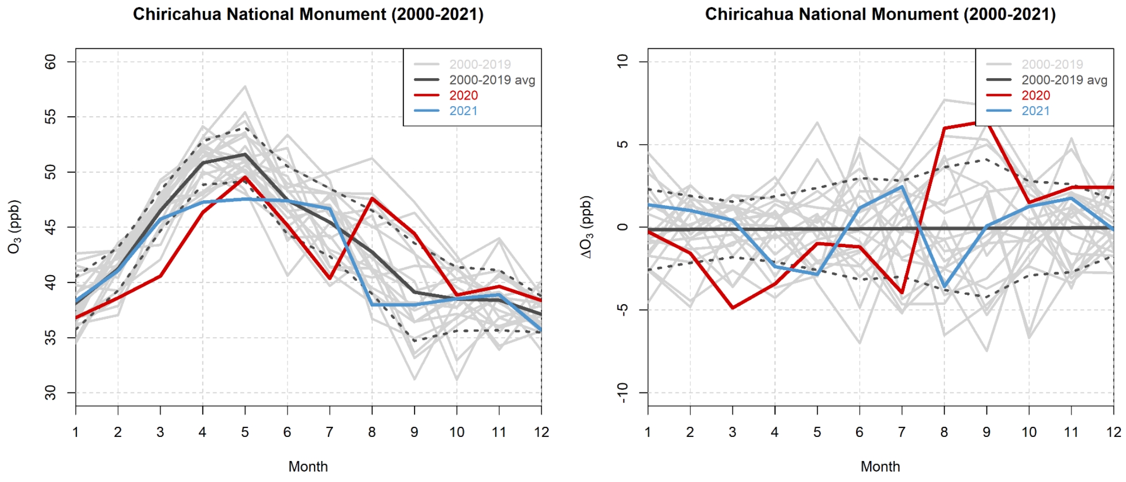

Figure S10. Same as Fig. S8, but for CNM.

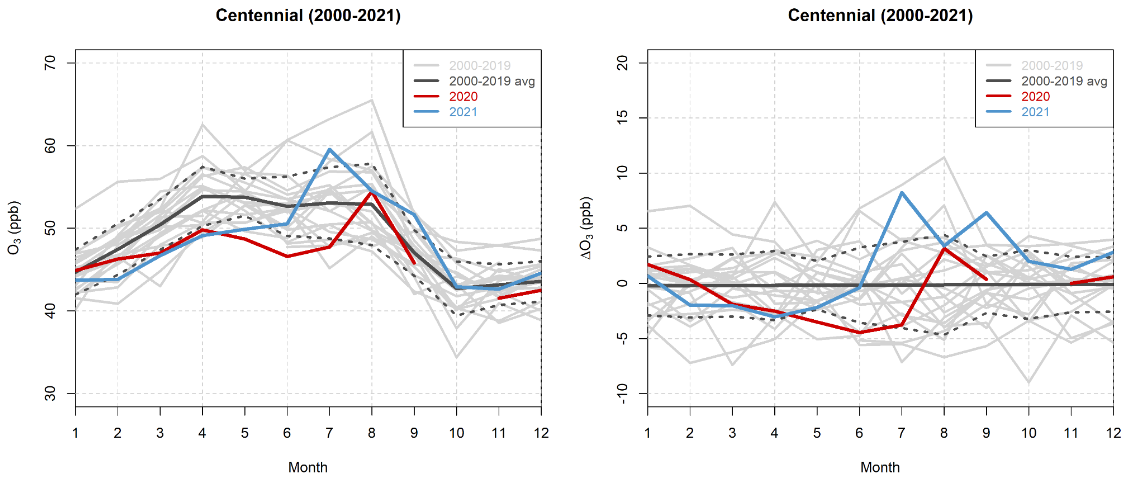

Figure S11. Same as Fig. S8, but for CNR.

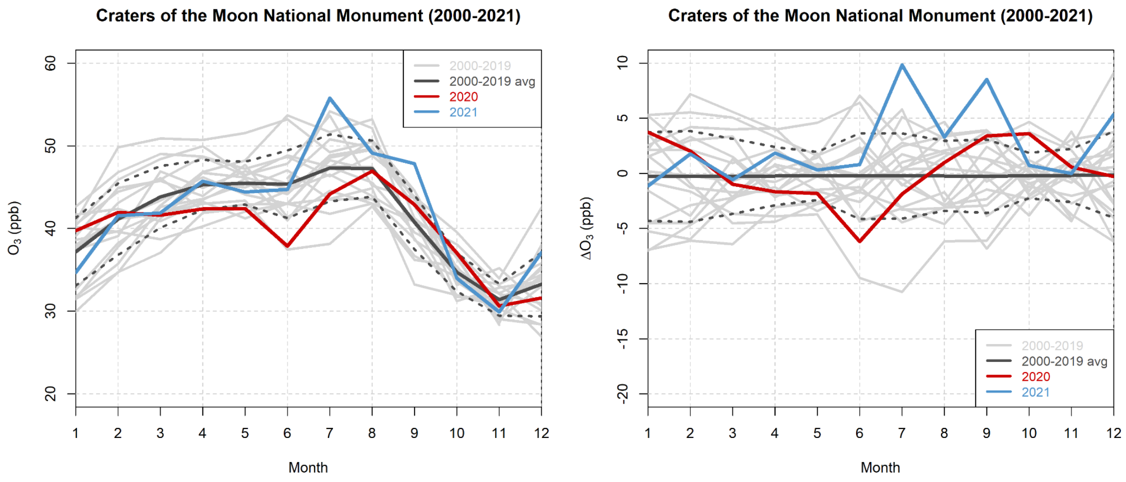

Figure S12. Same as Fig. S8, but for CRA.

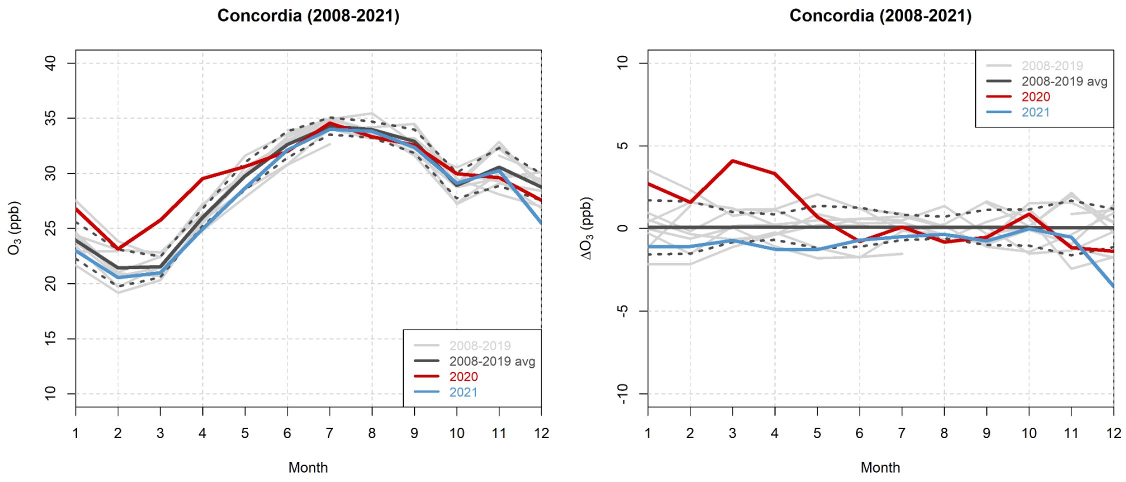

Figure S13. Same as Fig. S8, but for DCC.

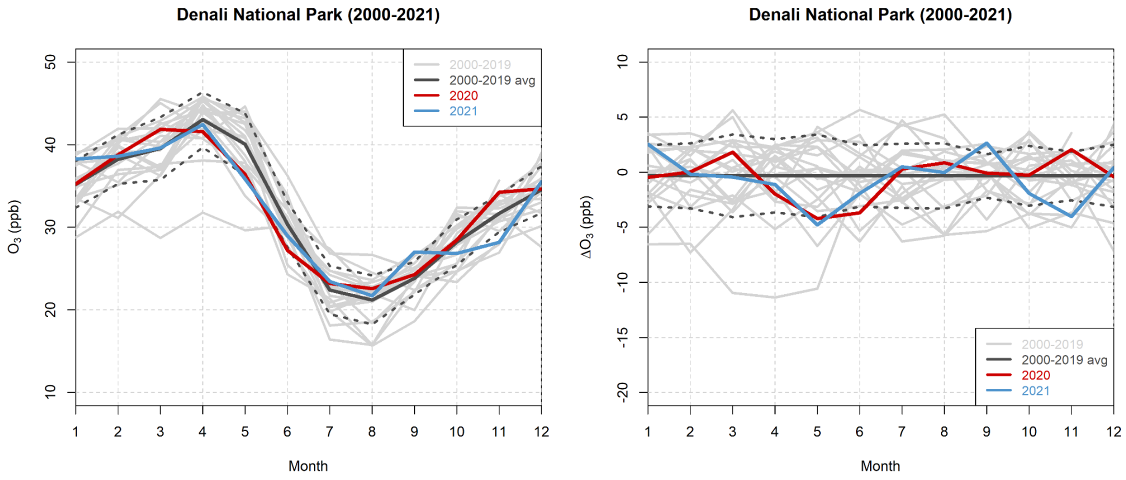

Figure S14. Same as Fig. S8, but for DEN.

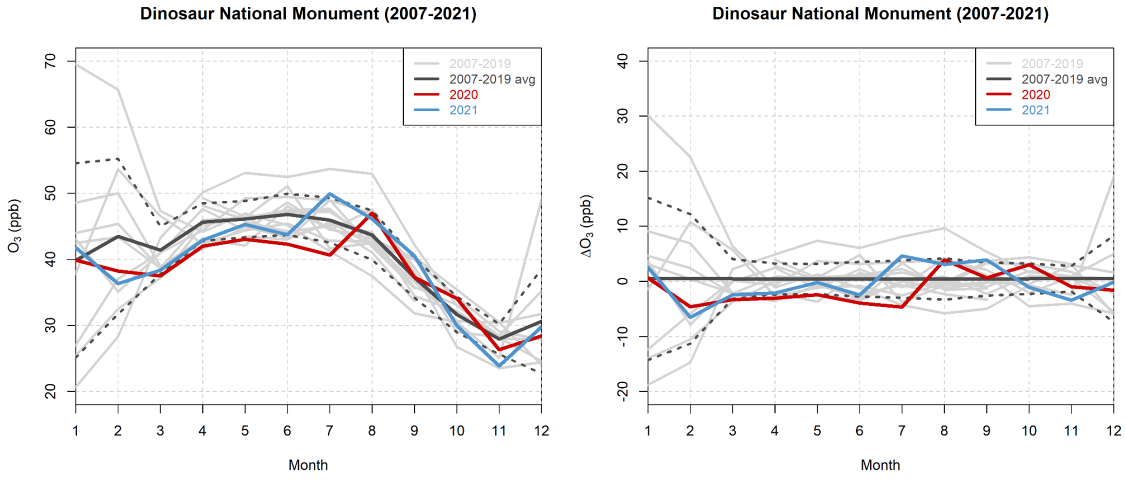

Figure S15. Same as Fig. S8, but for DIN.

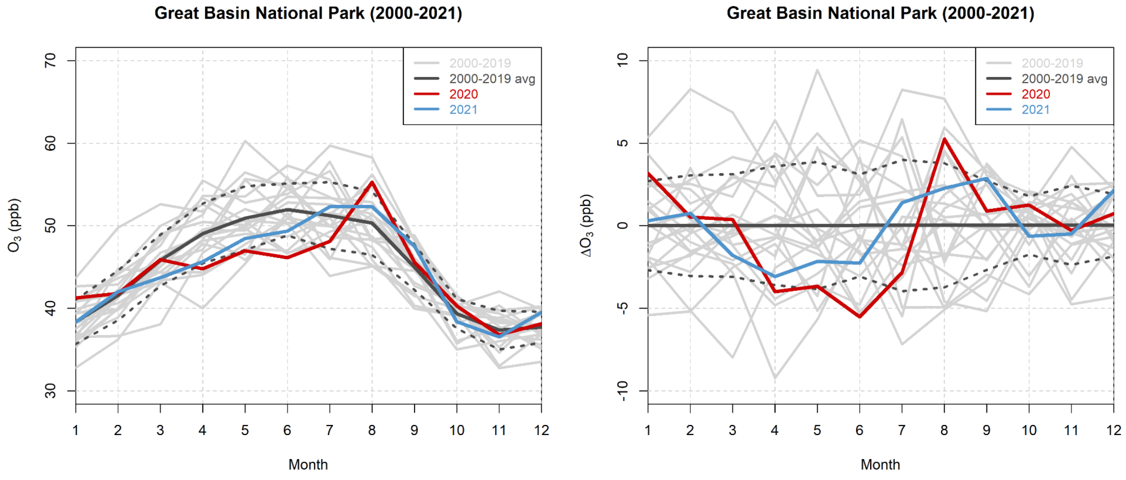

Figure S16. Same as Fig. S8, but for GBN.

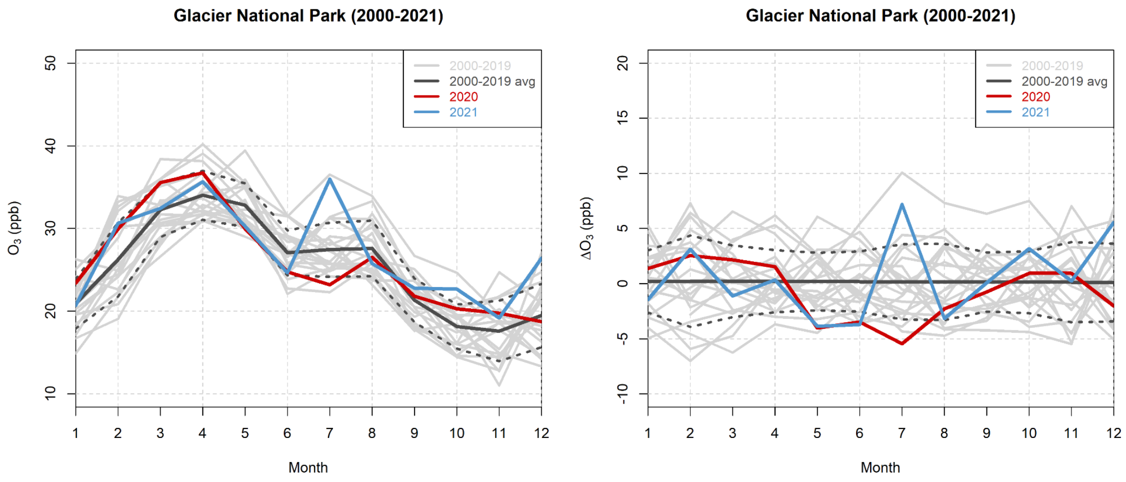

Figure S17. Same as Fig. S8, but for GNP.

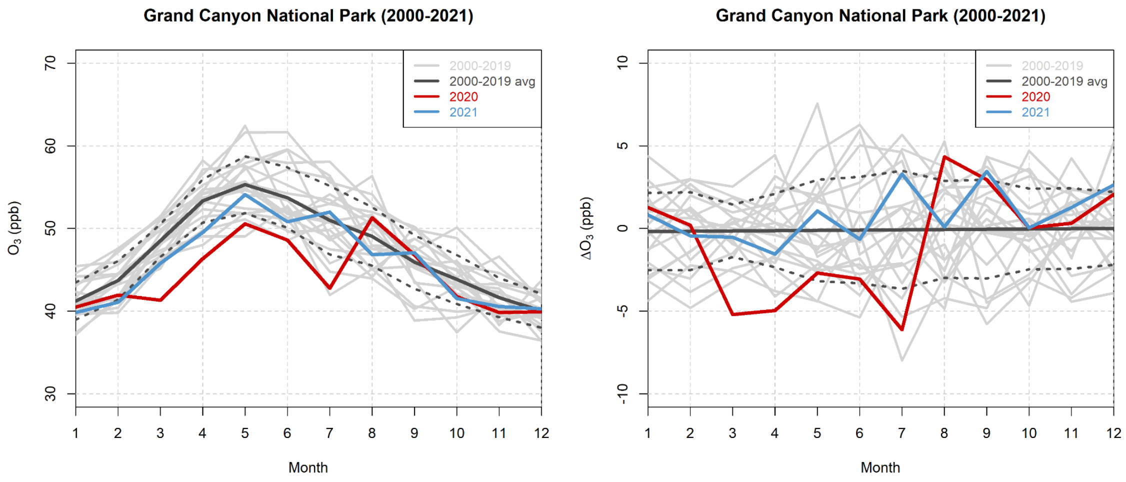

Figure S18. Same as Fig. S8, but for GRC.

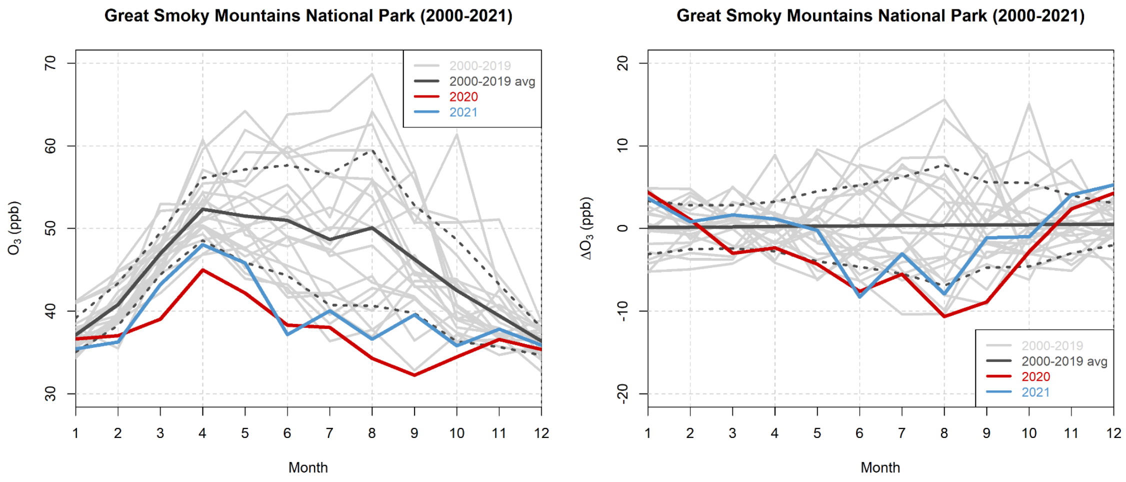

Figure S19. Same as Fig. S8, but for GSM.

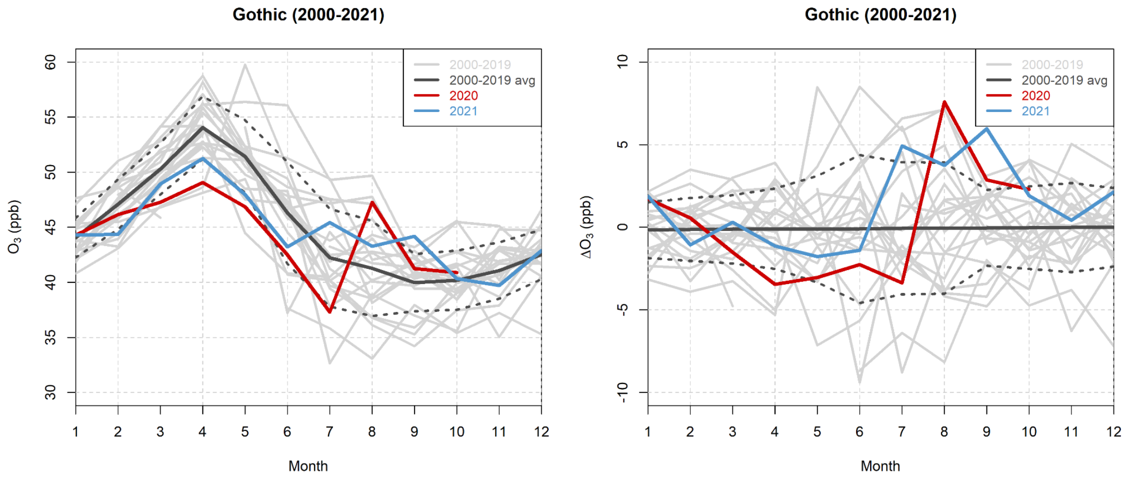

Figure S20. Same as Fig. S8, but for GTH.

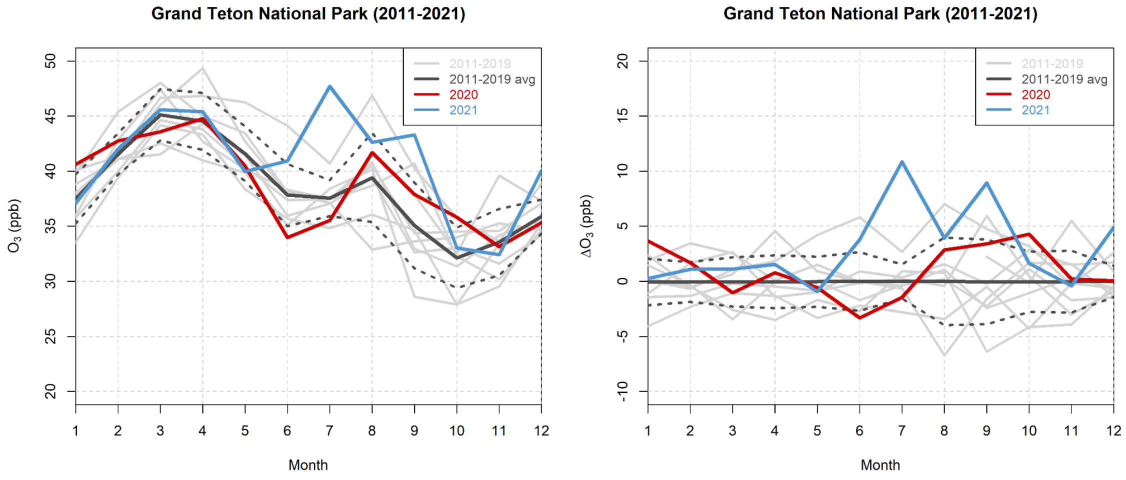

Figure S21. Same as Fig. S8, but for GTP.

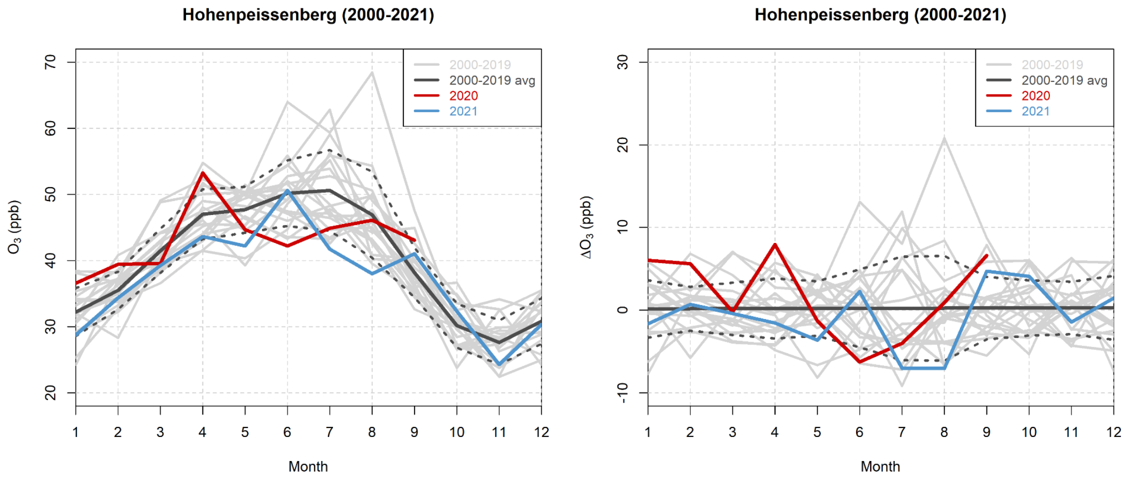

Figure S22. Same as Fig. S8, but for HPB.

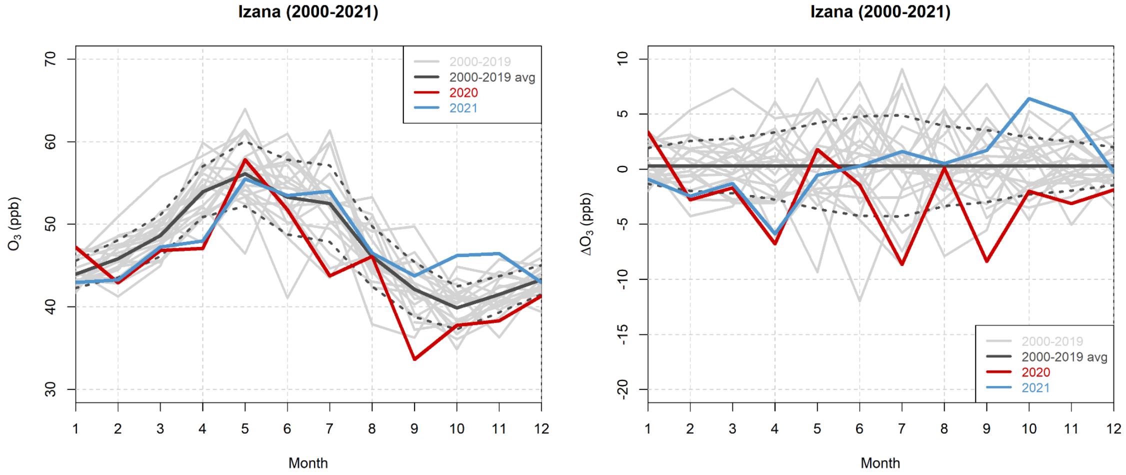

Figure S23. Same as Fig. S8, but for IZO.

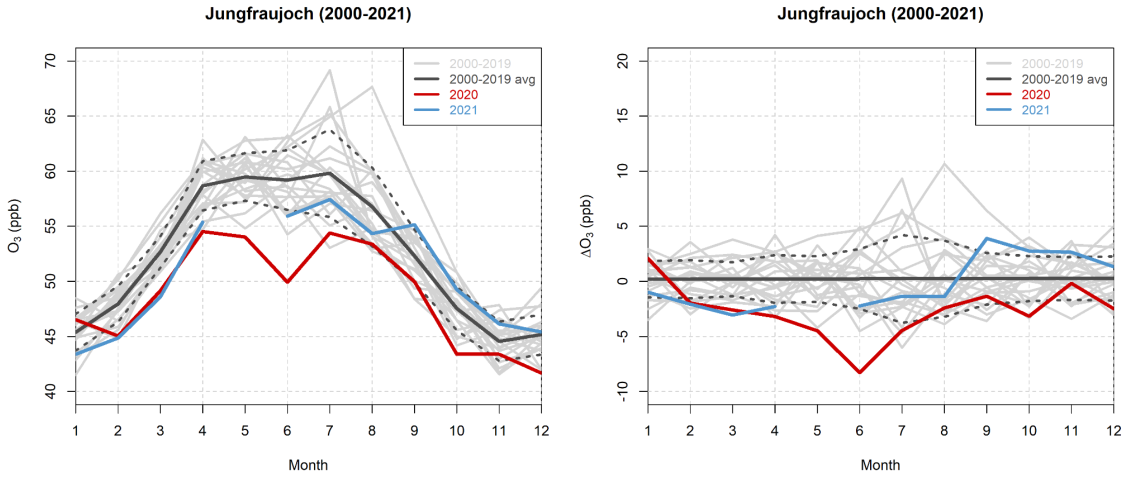

Figure S24. Same as Fig. S8, but for JFJ.

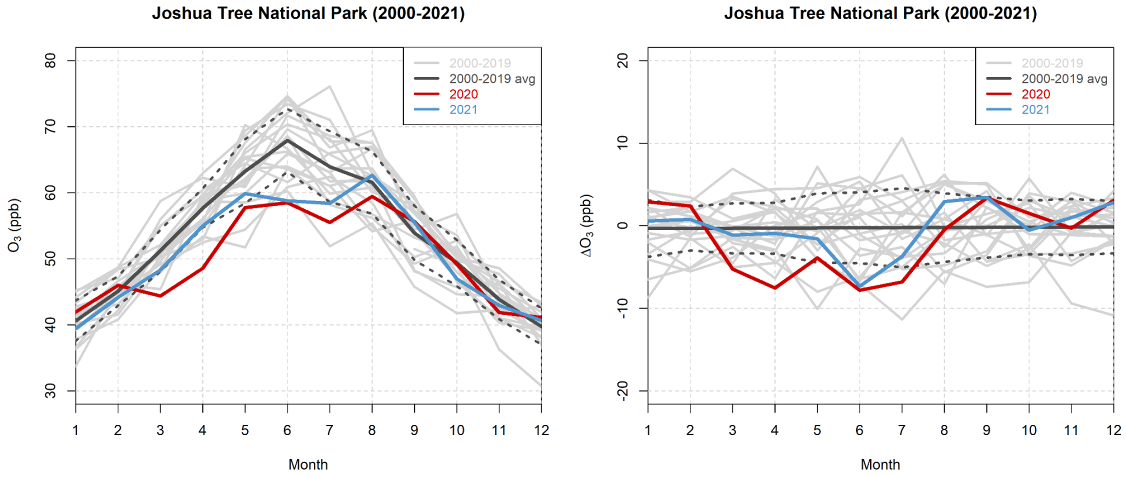

Figure S25. Same as Fig. S8, but for JOT.

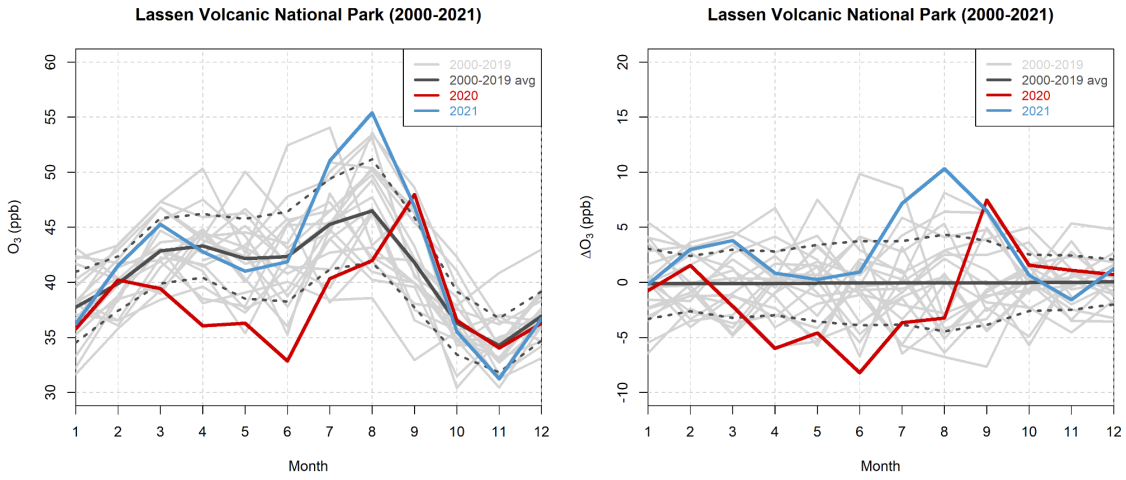

Figure S26. Same as Fig. S8, but for LAV.

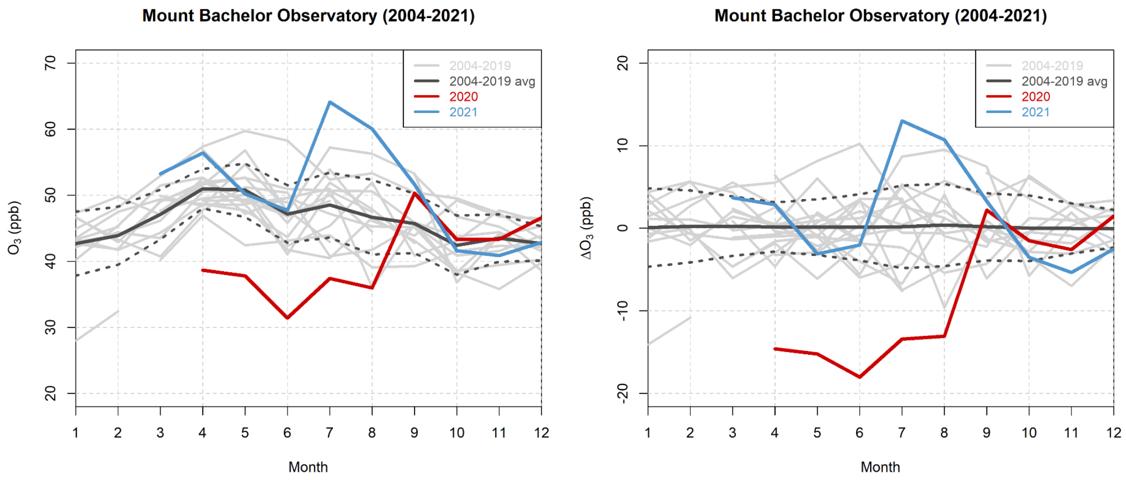

Figure S27. Same as Fig. S8, but for MBO.

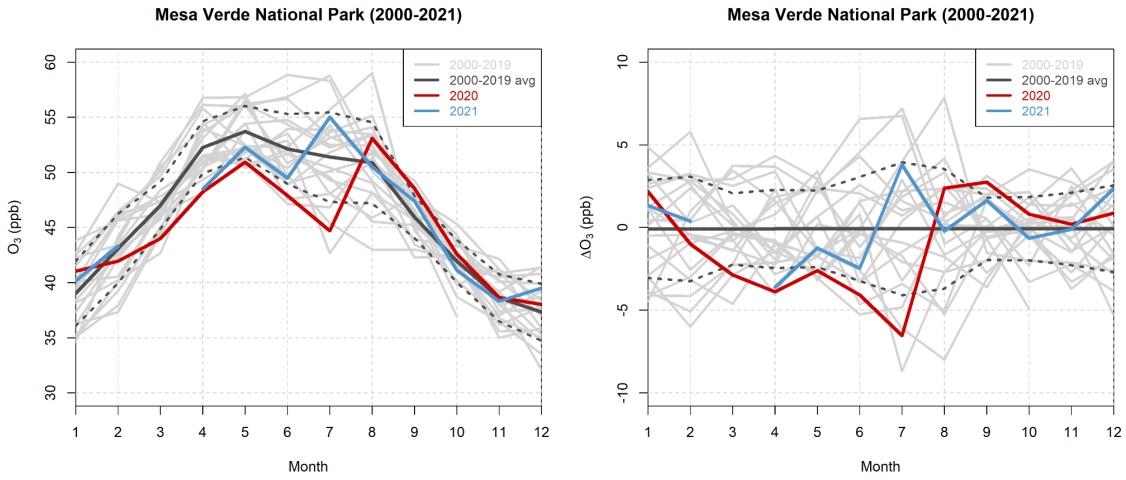

Figure S28. Same as Fig. S8, but for MEV.

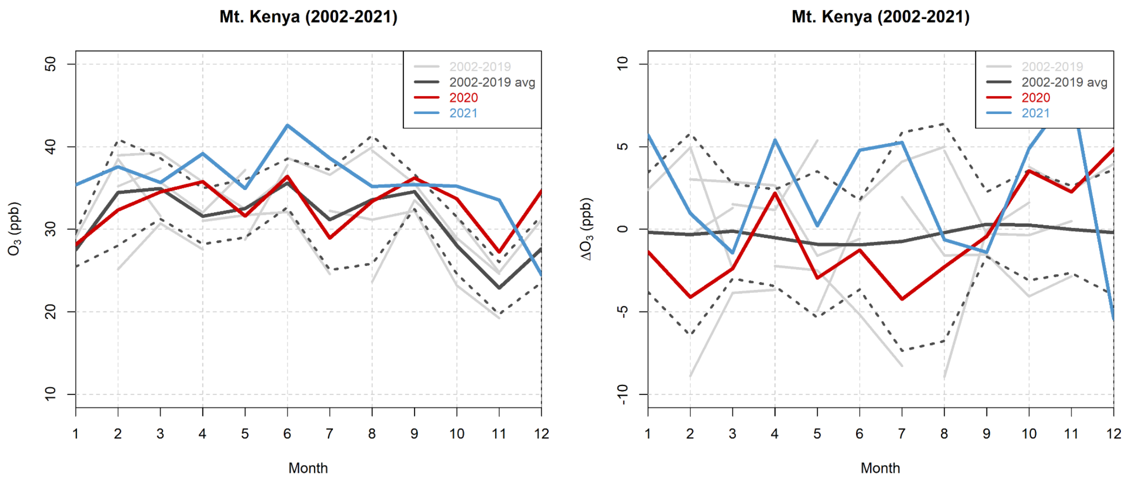

Figure S29. Same as Fig. S8, but for MKN.

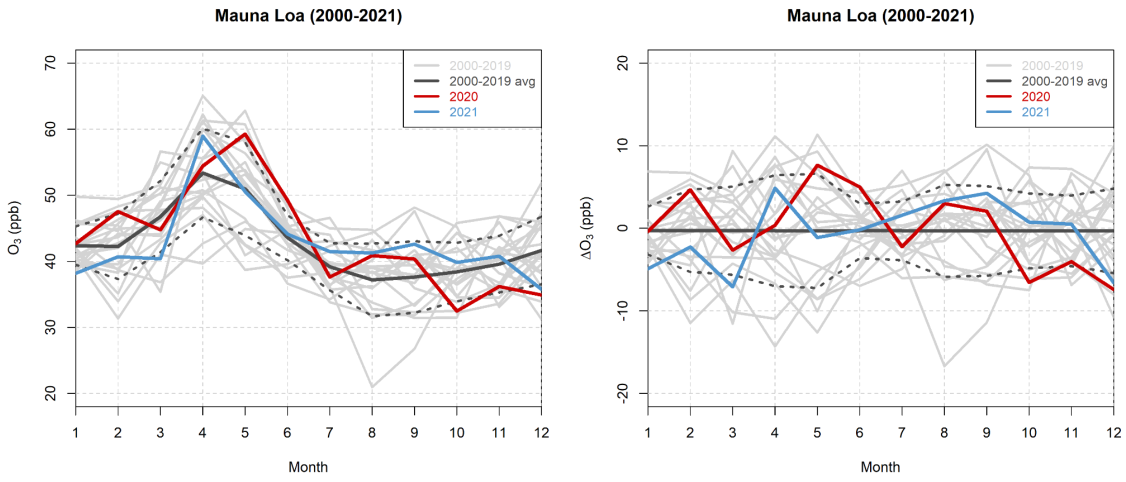

Figure S30. Same as Fig. S8, but for MLO.

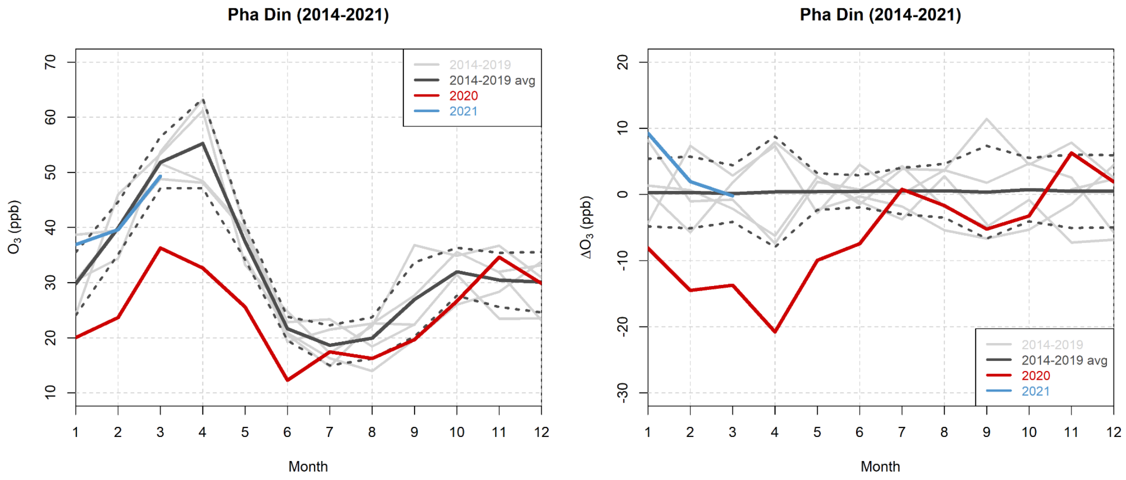

Figure S31. Same as Fig. S8, but for PDI.

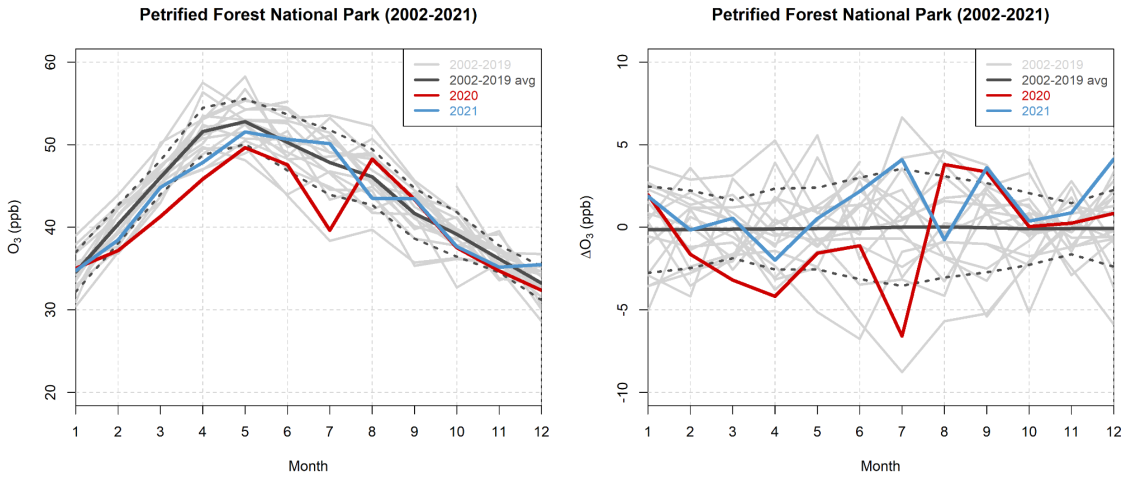

Figure S32. Same as Fig. S8, but for PFN.

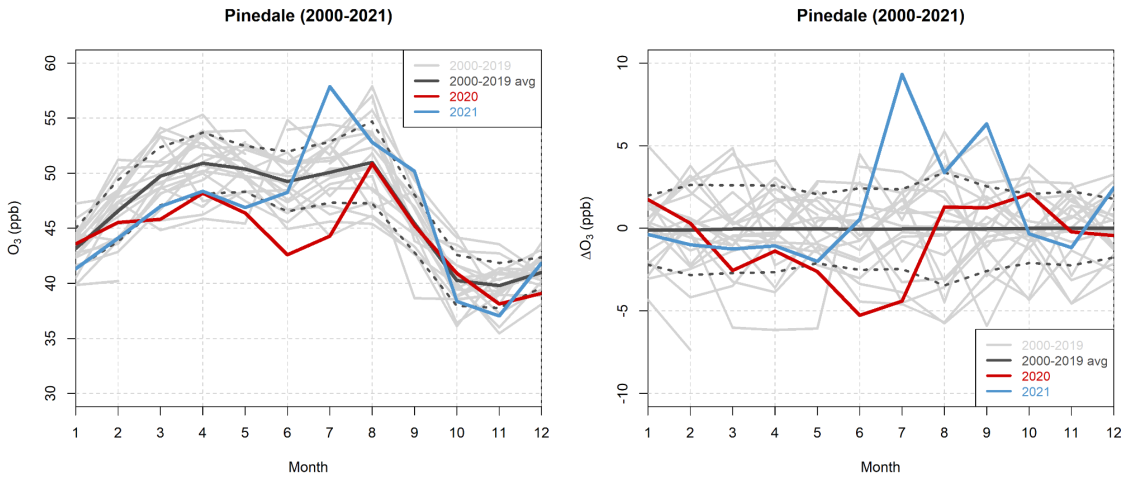

Figure S33. Same as Fig. S8, but for PND.

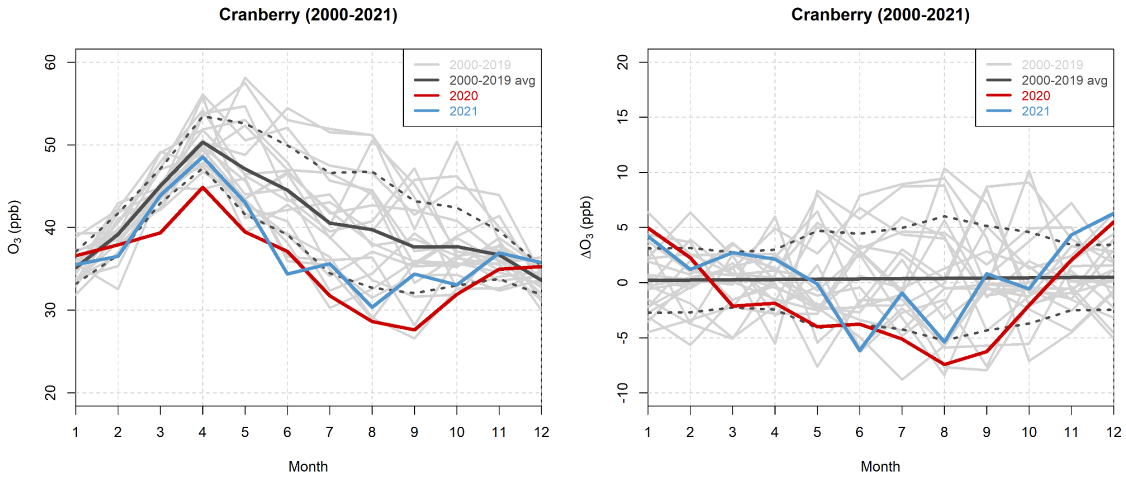

Figure S34. Same as Fig. S8, but for PNF.

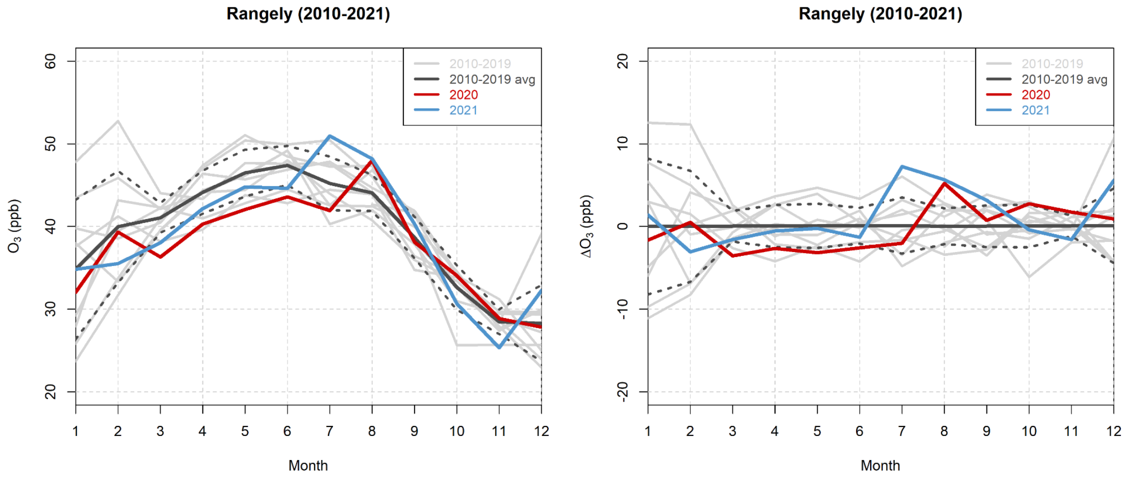

Figure S35. Same as Fig. S8, but for RAN.

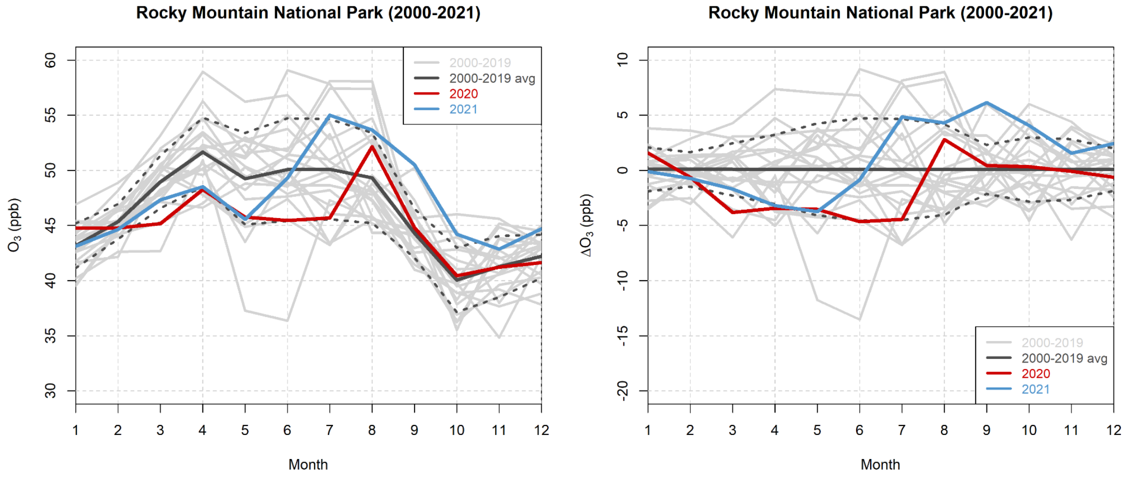

Figure S36. Same as Fig. S8, but for RMN.

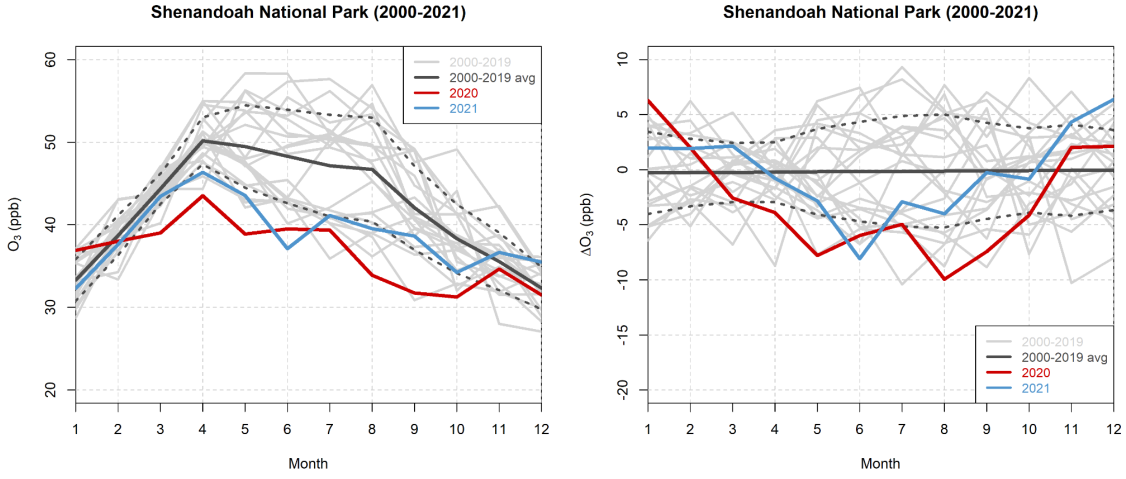

Figure S37. Same as Fig. S8, but for SHN.

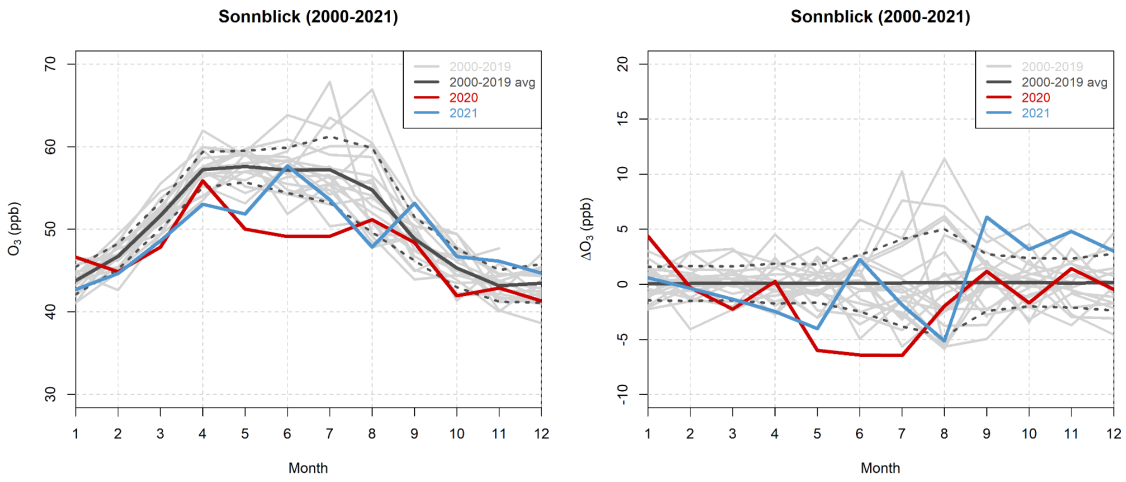

Figure S38. Same as Fig. S8, but for SNB.

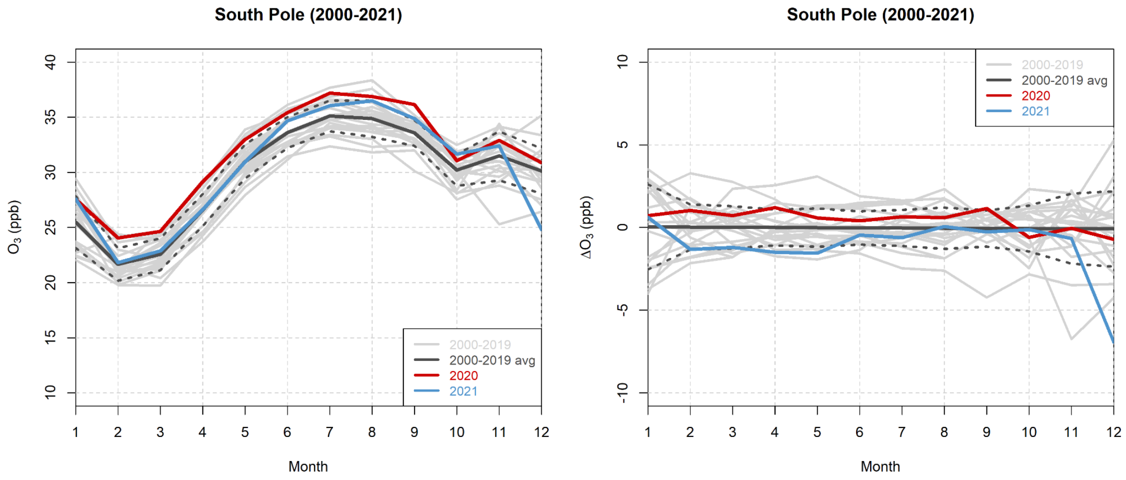

Figure S39. Same as Fig. S8, but for SPO.

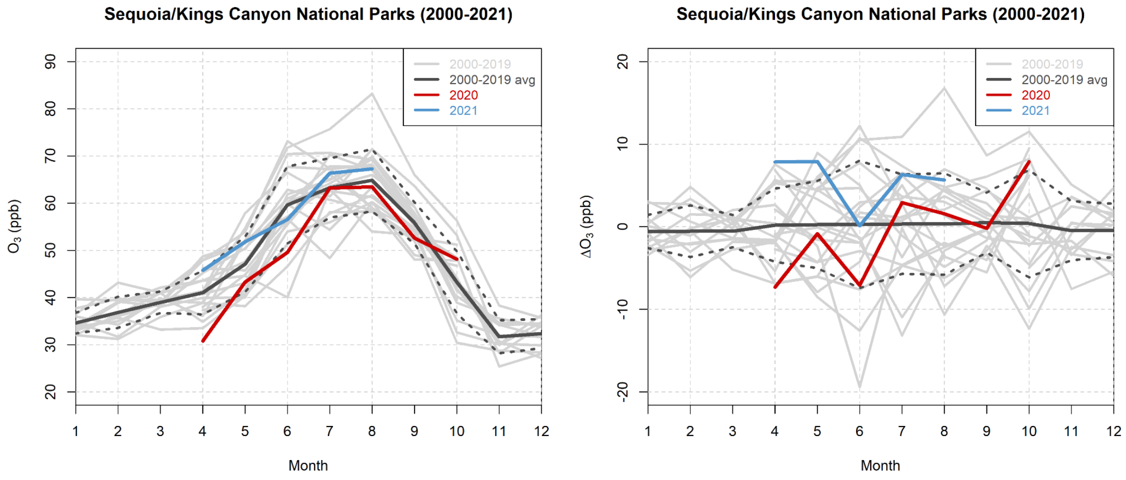

Figure S40. Same as Fig. S8, but for SQA.

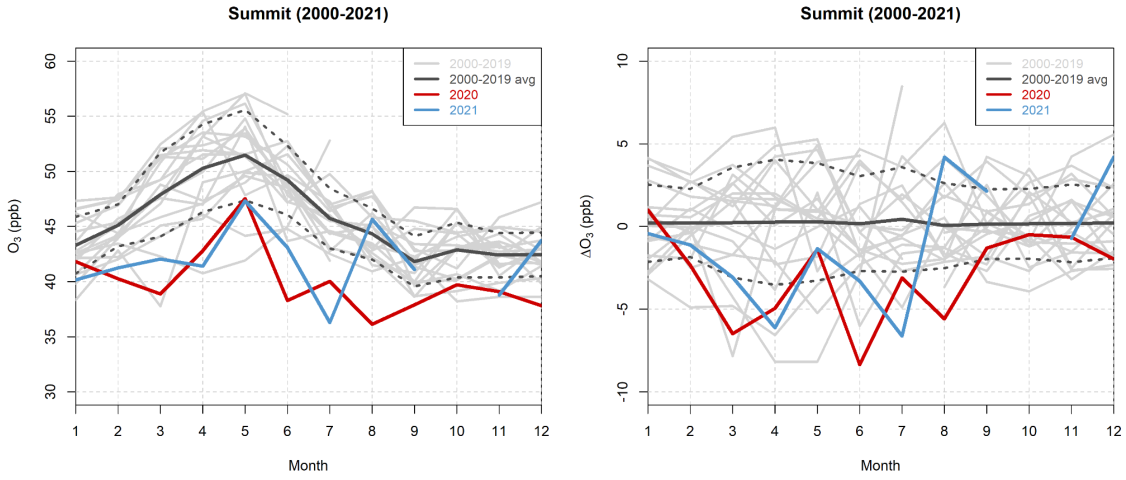

Figure S41. Same as Fig. S8, but for SUM.

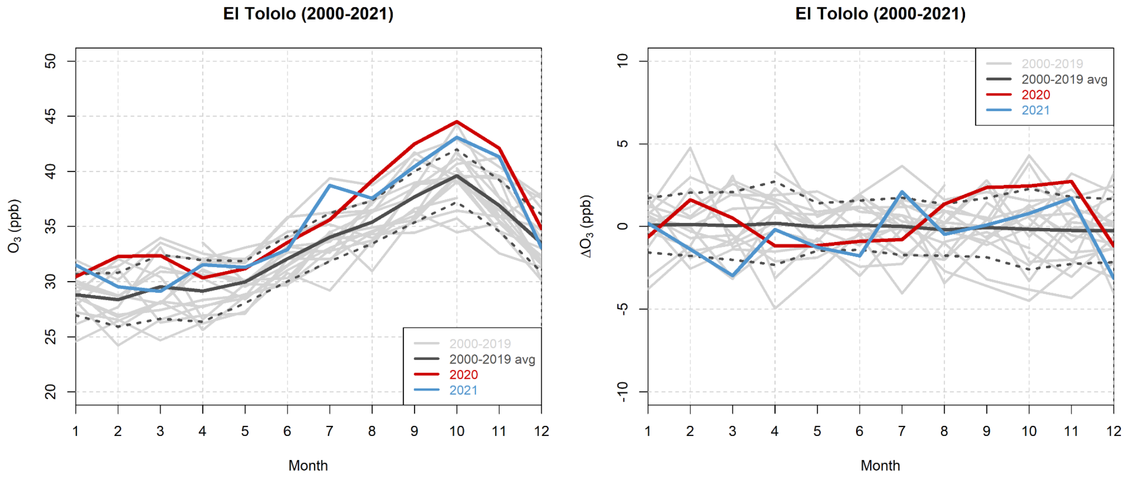

Figure S42. Same as Fig. S8, but for TLL.

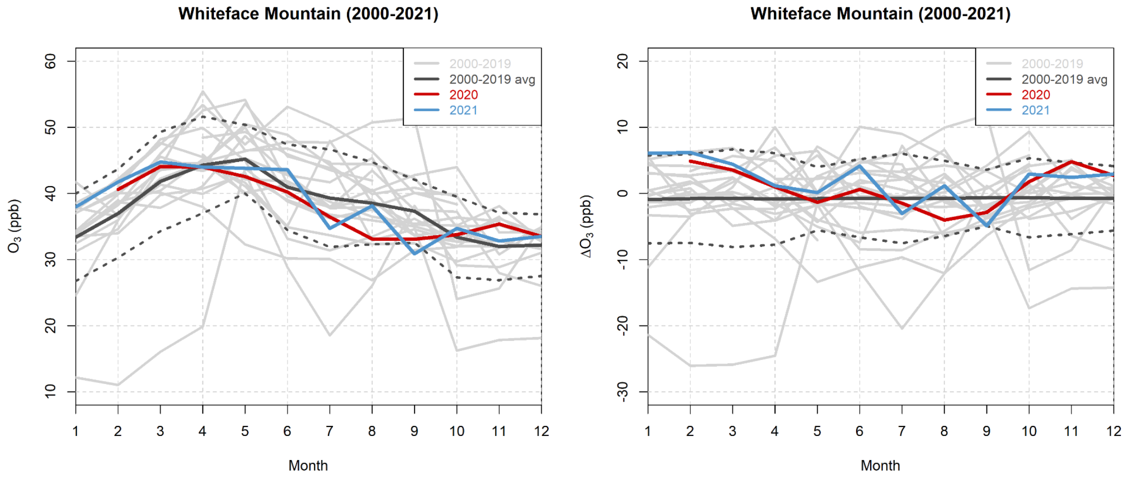

Figure S43. Same as Fig. S8, but for WFM.

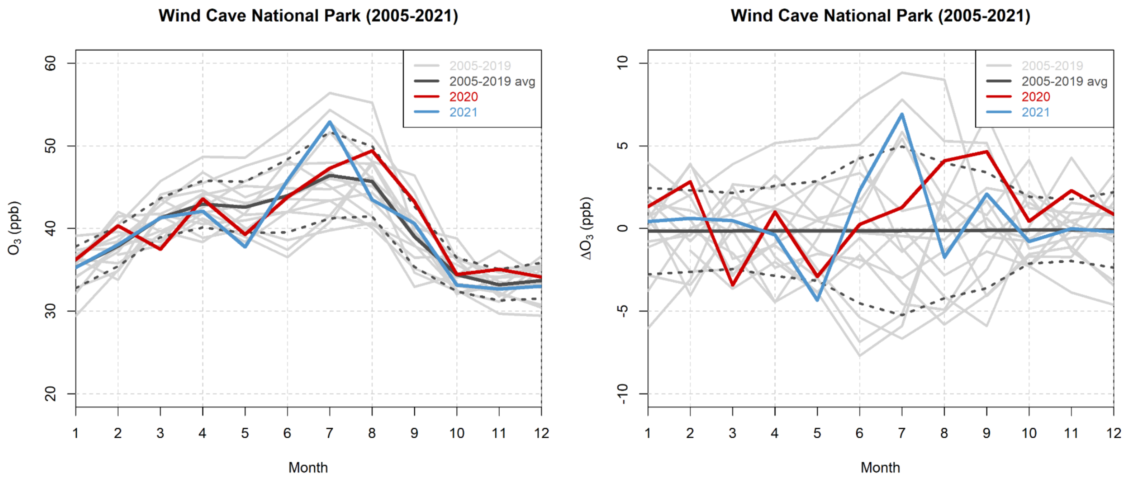

Figure S44. Same as Fig. S8, but for WNC.

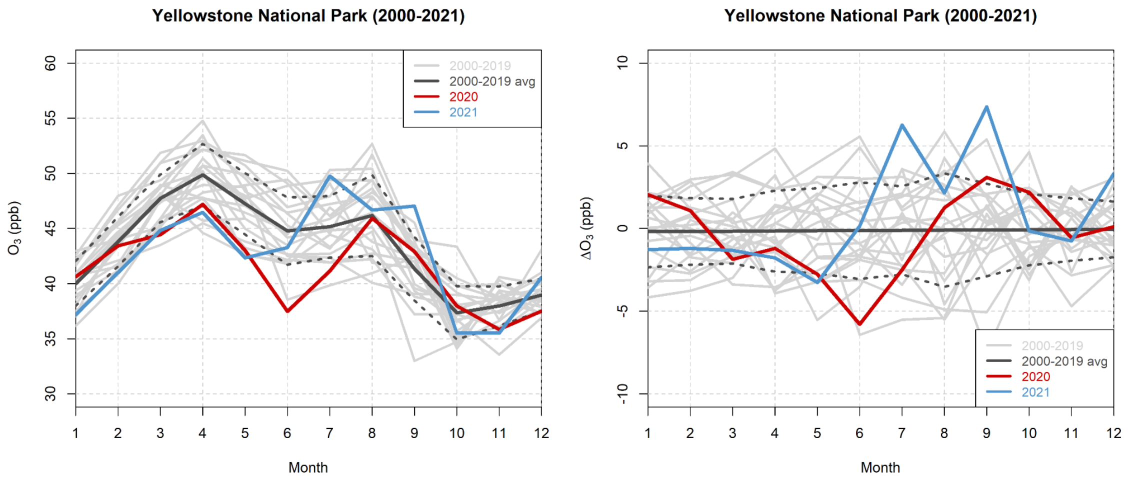

Figure S45. Same as Fig. S8, but for YEL.

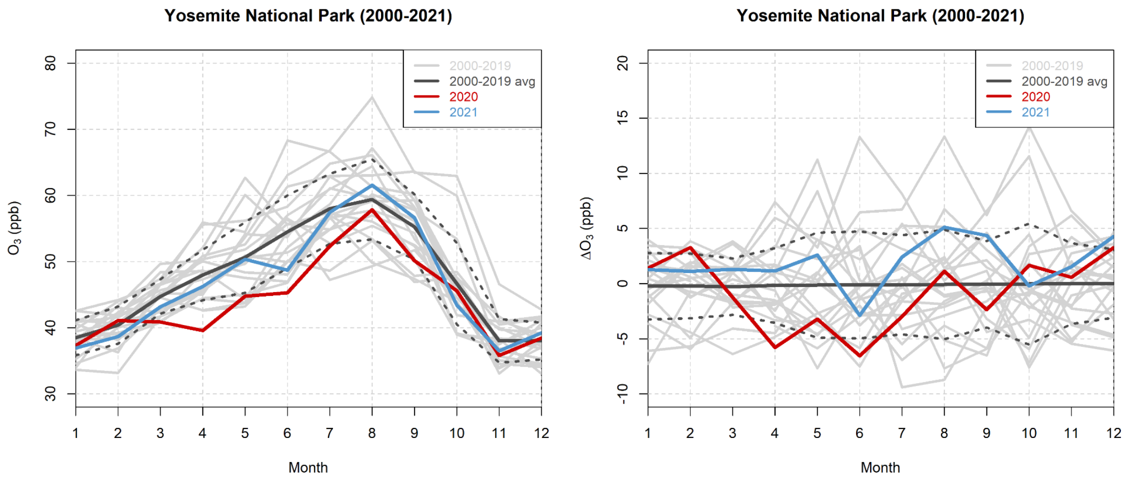

Figure S46. Same as Fig. S8, but for YOS.

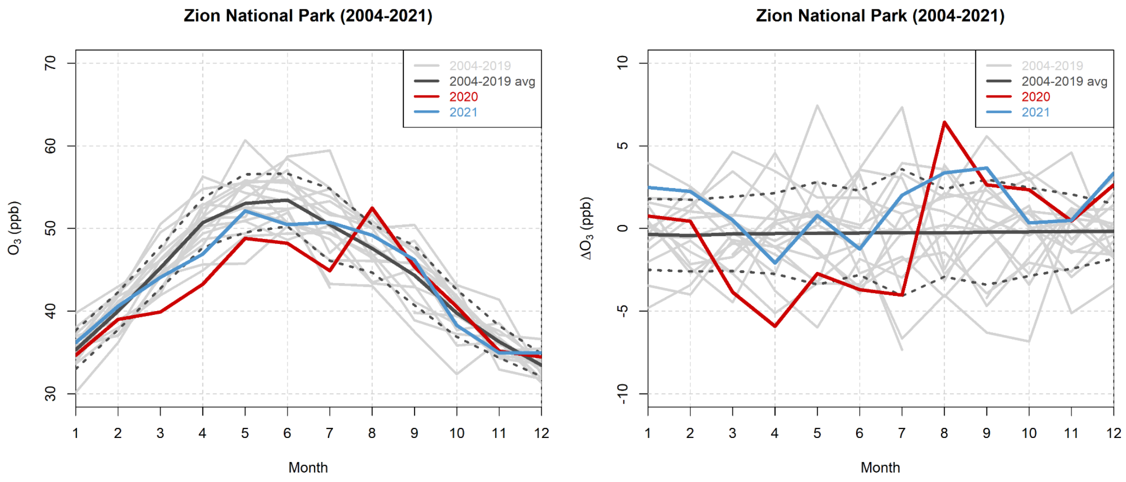

Figure S47. Same as Fig. S8, but for ZIO.

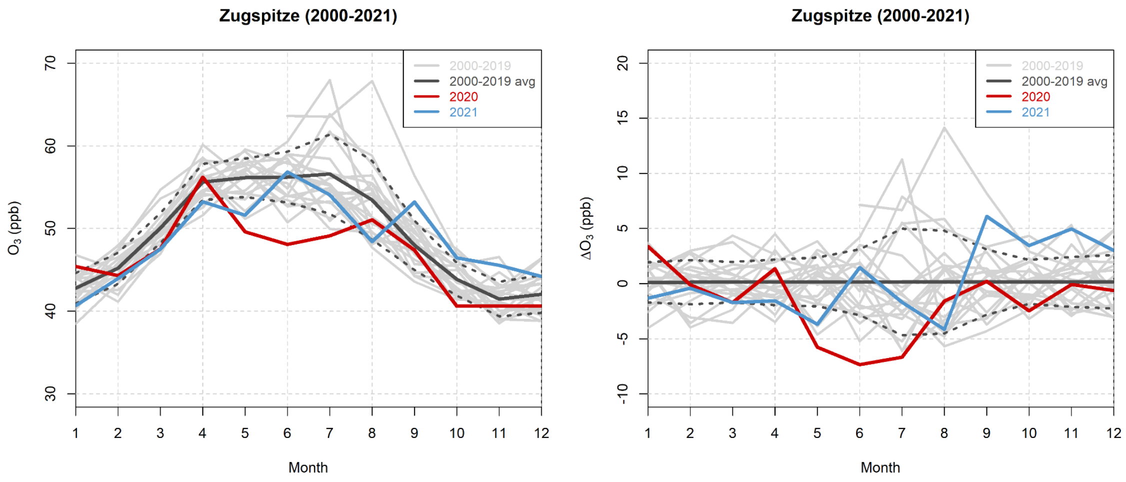

Figure S48. Same as Fig. S8, but for ZSF.
